# Diversity and Within-Host Evolution of Leishmania donovani from Visceral Leishmaniasis Patients with and without HIV Coinfection in Northern Ethiopia

**DOI:** 10.1128/mBio.00971-21

**Published:** 2021-06-29

**Authors:** Susanne U. Franssen, Yegnasew Takele, Emebet Adem, Mandy J. Sanders, Ingrid Müller, Pascale Kropf, James A. Cotton

**Affiliations:** a Wellcome Sanger Institute, Hinxton, United Kingdom; b Leishmaniasis Research and Treatment Centre, University of Gondar, Gondar, Ethiopia; c Department of Infectious Disease, Imperial College London, London, United Kingdom; Albert Einstein College of Medicine

**Keywords:** *Leishmania*, genomics, host-parasite relationship, molecular epidemiology, population genetics

## Abstract

Visceral leishmaniasis (VL) is a fatal disease and a growing public health problem in East Africa, where Ethiopia has one of the highest VL burdens. The largest focus of VL in Ethiopia is driven by high prevalence in migrant agricultural workers and associated with a high rate of coinfection with HIV. This coinfection makes VL more difficult to treat successfully and is associated with a high rate of relapse, with VL/HIV patients frequently experiencing many relapses of VL before succumbing to this infection. We present genome-wide data on Leishmania donovani isolates from a longitudinal study of cohorts of VL and VL/HIV patients reporting to a single clinic in Ethiopia. Extensive clinical data allow us to investigate the influence of coinfection and relapse on the populations of parasites infecting these patients. We find that the same parasite population is responsible for both VL and VL/HIV infections and that, in most cases, disease relapse is caused by recrudescence of the population of parasites that caused primary VL. Complex, multiclonal infections are present in both primary and relapse cases, but the infrapopulation of parasites within a patient loses genetic diversity between primary disease presentation and subsequent relapses, presumably due to a population bottleneck induced by treatment. These data suggest that VL/HIV relapses are not caused by genetically distinct parasite infections or by reinfection. Treatment of VL does not lead to sterile cure, and in VL/HIV, the infecting parasites are able to reestablish after clinically successful treatment, leading to repeated relapse of VL.

## INTRODUCTION

Leishmaniasis is a major neglected tropical disease, with the most severe form—visceral leishmaniasis (VL)—being the second most deadly parasitic disease, responsible for 20,000 to 40,000 deaths per year (1) together with significant morbidity (loss of >2 million disability-adjusted life years [DALYs] [2]). VL is caused by infections with protozoan parasites from the Leishmania donovani species complex. While clinical VL is frequently fatal without adequate treatment, most L. donovani infections remain asymptomatic (see reviews in references 3 and 4), or at least subclinical, and much remains unknown about what leads to the development of severe disease (5) or to the success or failure of treatment. Most of the global burden of VL is in the Indian subcontinent, but East Africa represents the second largest burden of VL caused by L. donovani. While the number of cases has been falling in the Indian subcontinent (6), they continue to increase in Africa. Ethiopia, Sudan, and South Sudan have the highest prevalence in this region, and VL is one of the most significant vector-borne diseases in Ethiopia, with >3 million people at risk of infection in a number of distinct foci in lowland areas of the country (7, 8).

The most important focus of VL in Ethiopia is around Metema and Humera in the Northwest of the country, where since the 1970s, there has been an important focus of disease particularly associated with seasonal migration of nonimmune laborers from the surrounding non-endemic highland regions for agricultural work. Several outbreaks have occurred in this focus, and there is evidence that this focus is spreading and has seeded outbreaks in other previously non-endemic areas (7–10). One factor in the growth of VL in Ethiopia is coinfection and, particularly, coinfection of L. donovani with HIV. While VL/HIV coinfection is a widespread concern (11), Ethiopia has the highest rate of VL/HIV coinfections in Africa, and possibly globally; while estimates vary between studies, HIV may be present in >20% of VL cases (7, 12). In Ethiopia and elsewhere, HIV coinfection is known to increase the probability that L. donovani infections progress to symptomatic visceral leishmaniasis (13, 14). These coinfections are difficult to treat, with poor clinical outcomes for patients with very high mortality and high rates of relapse following treatment (14–18). It is generally thought that cure of VL is not sterile, so that parasites continue to be present, but there is little direct evidence of this. It is unknown whether relapses of VL are due to reinfection by parasites, which we would expect to occur more commonly when patient immunity is compromised, or due to recrudescence of the original infection from parasites that persist through original treatment.

There has been significant work on the genetics of L. donovani in East Africa, which has revealed Ethiopia as a hot spot for diversity of visceral leishmaniasis, with two major genetic groups of L. donovani occurring (19). These groups were shown to be transmitted by distinct sandfly vectors and are separated by the rift valley (20, 21). A single Ethiopian isolate has been identified from a third group of parasites, related to those found on the Arabian Peninsula and adjacent areas of the Middle East (22). To add to this complex picture, a 2004 outbreak of VL in northern Ethiopia has been associated with a diverse population of parasites that persisted after the outbreak (9), some of which are hybrids between two of these groups (19, 23, 24). However, the studies to date represent relatively small numbers of isolates often collected over an extended period of time and from a wide geographical area. While this has established an important baseline in understanding the diversity and basic structure of parasite populations, these studies give little or no insight into the origins of the observed genetic variation or its phenotypic consequences. For example, any genetic variation driving variation in disease presentation or clinical outcome is confounded by variation over time and space.

Here, we report whole-genome sequence data from parasites isolated from two cohorts, VL patients and patients coinfected with HIV and *Leishmania*. All patients were recruited at a single clinic in Northern Ethiopia over a period of 18 months. This makes our work one of the largest studies of genome-wide genetic variation in leishmaniasis at a single place and time. Extensive clinical and immunological data were collected from these patients over 12 months of follow-up, and long-term outcomes were recorded up to 3 years post-VL diagnosis; thus, the parasite genome data we report is from a well-understood patient population. Full details of patient recruitment and clinical data from these patients were previously reported (25). Parasite isolation is generally only possible when either bone marrow or splenic aspirates are being collected for parasitological investigation, which is routinely performed at the time of diagnosis of each VL episode. This includes additional isolates from individual patients that were collected where possible during subsequent episodes of clinical relapse during the follow-up of this cohort. We thus present the first—to our knowledge—longitudinal data on within-host evolution of *Leishmania* parasites. These data demonstrate that most relapse cases are caused by the originally infecting parasites and that the parasite infrapopulations (the population of parasites within a host at a particular time) undergo strong bottlenecks during treatment. VL relapse is not generally associated with known markers for drug resistance, and despite some diversity, we show that parasites from VL infections without HIV and VL/HIV coinfections represent the same parasite population. We conclude that relapse in VL/HIV patients is due to the persistence of parasites after treatment that, in the absence of fully functional host immunity, can re-expand to cause the recurrent episodes of disease.

## RESULTS

### No distinct phylogenetic origin of parasites from VL and VL/HIV patients in northern Ethiopia.

We sequenced the genomes of 108 L. donovani isolates from a total of 98 VL patients in Ethiopia. For five isolates, two aliquots of the same initial culture were processed and sequenced independently. The genome-wide haploid coverage of the sequenced samples ranged between 7 and 30 (median, 13). Our study included 68 patients who had VL only and 29 patients who were additionally coinfected with HIV and were receiving antiretroviral treatment (25). The complete metadata of samples taken, technical replicates, and patients are summarized in Table S1 in the supplemental material.

10.1128/mBio.00971-21.9 TABLE S1 Summary of all isolates in this study. Download Table S1, XLSX file, 23 KB. Copyright © 2021 Franssen et al. 2021 Franssen et al. https://creativecommons.org/licenses/by/4.0/ This content is distributed under the terms of the Creative Commons Attribution 4.0 International license.

**FIG 1:**
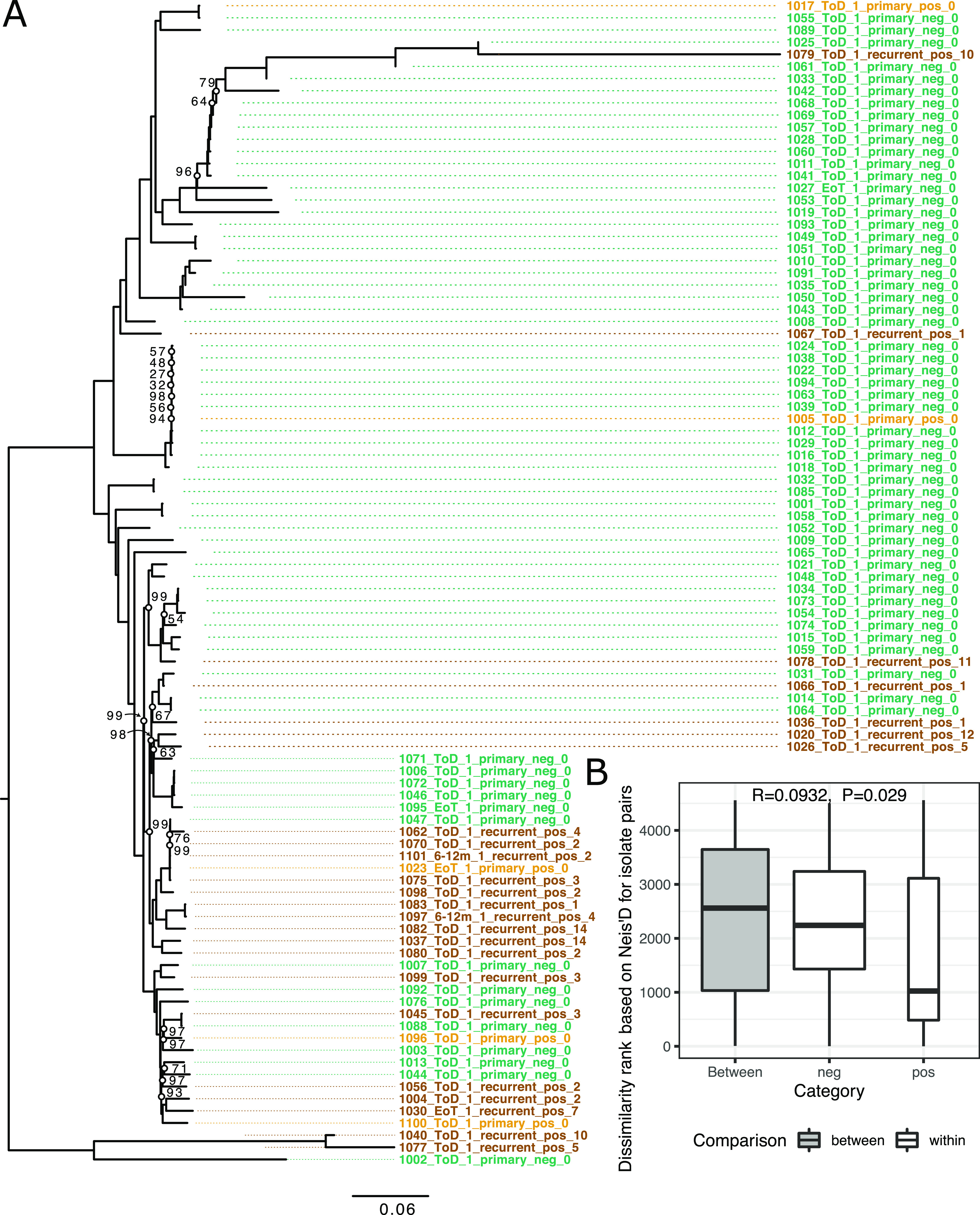
Genomic relatedness between first parasite isolates taken from each patient. (A) Phylogeny of first isolate taken from each patient. Parasite sample names are encoded, including the patient number, treatment status, patient isolate count, VL type, HIV status, and the VL episode number of the patient the isolate was taken. Sample color indicates VL and HIV status: green for primary VL episodes in HIV-negative patients, light orange for primary VL episodes in HIV-positive patients, and ochre for samples from VL relapse in VL/HIV-coinfection cases. Bootstrap values are indicated at branch nodes marked with open circles. All other nodes had 100% bootstrap support. The phylogeny is rooted based on the inclusion of an L. infantum outgroup (data not shown). (B) ANOSIM results comparing the sum of ranked pairwise genetic distances (Nei’s D) within and between HIV-positive and -negative samples shown in the phylogeny.

These parasites were isolated from a cohort of VL patients in which 78.1% of the successfully treated VL/HIV patients relapsed within a 3-year time period, while none of the patients without HIV infection relapsed (25). Therefore, we tested for the possibility that there is a genetic difference between parasites isolated from HIV-negative versus HIV-positive VL patients that could explain this difference in relapse rate. Phylogenetic reconstruction using the first parasite isolate taken from each patient, which is not necessarily the first episode of VL in the HIV patients, showed the genetic relatedness between isolates based on whole-genome single nucleotide polymorphism (SNP) data (Fig. 1A). It suggested that there was no clear phylogenetic structure between parasites from the two groups. To investigate more subtle genetic structure, we used two measures to test for a possible association of HIV status with genome-wide parasite genomics, based on those isolates from 67 HIV-negative and 29-HIV positive patients, respectively. Analysis of similarities (ANOSIM) using pairwise genome-wide Nei’s D as distance measure (*R* = 0.09321, *P* value = 0.0290, false-discovery rate [FDR] = 0.048) as well as Blomberg’s *K* (*K* = 0.0348, *P* value = 0.048, FDR = 0.048) both showed an association of HIV status with the phylogeny at an α of 0.05 but not at an α of 0.01. Isolates from HIV-positive patients were more frequently samples from second or subsequent relapses of VL disease, which could introduce a bias due to a longer period of within-host evolution in these infections. Therefore, we also tested for the association of HIV status based on isolates taken during the first episode of VL disease for a particular patient (see Fig. S1) (67 samples from HIV-negative and 5 samples from HIV-positive patients). In this case, no significant association was present (ANOSIM, *R* = −0.0602, *P* value = 0.6; Blomberg’s *K*, *K* = 0.0197, *P* value = 0.4).

10.1128/mBio.00971-21.1 FIG S1 Genomic relatedness between first parasite isolates taken from each patient of primary VL only. Download FIG S1, PDF file, 0.05 MB. Copyright © 2021 Franssen et al. 2021 Franssen et al. https://creativecommons.org/licenses/by/4.0/ This content is distributed under the terms of the Creative Commons Attribution 4.0 International license.

### Recurrent VL in HIV patients is caused by persistent parasites.

**FIG 2:**
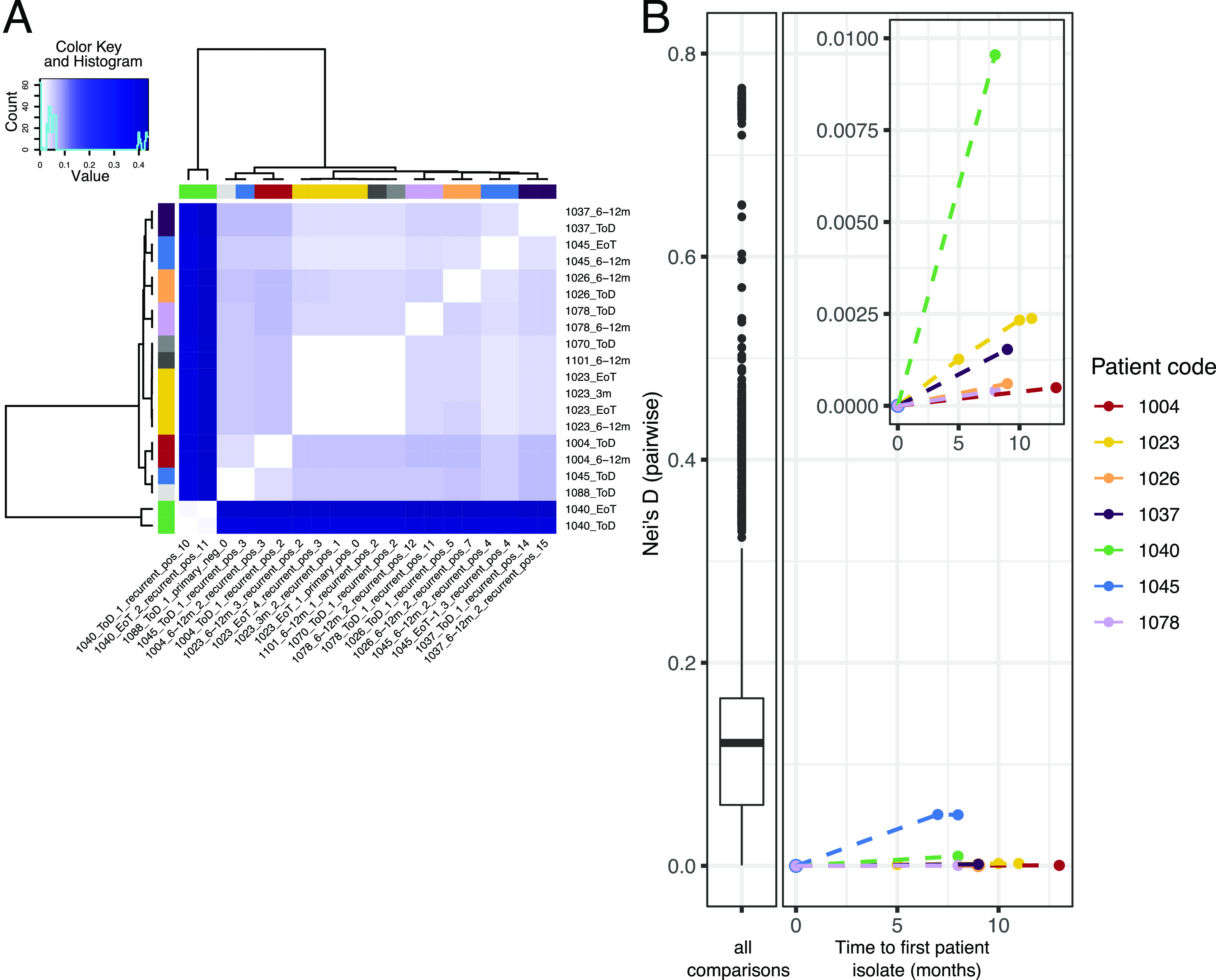
Recurrent VL is predominantly caused by persistent parasites, not reinfection. For seven patients, parasite isolates were taken at multiple time points during the course of their VL infection (Table 1). (A) Heat map of pairwise Nei's distances between isolates from the seven patients with longitudinal sampling along with any other isolates from our cohort most closely related to those. Row and column side colors on the outside indicate patient origin, with colors as used described for panel B, except that gray indicates isolates from patients represented by only a single isolate. Samples appear in the same order in row and column labels, but sample row names are abbreviated. (B) Increase of genetic distance between the first parasite isolate from a patient and subsequent isolates. (Left) Distribution of pairwise genetic distances across all 106 parasite isolates, excluding technical replicates and isolates with incomplete metadata. (Right) Pairwise Nei’s distances between the first isolate for each patient with longitudinal samples and subsequent samples from that patient (*y* axis), plotted against the time in months between sampling dates (*x* axis). Samples are colored by patient origin.

Seven of the patients in our study had multiple parasite isolates taken as part of clinical investigation of VL relapses during the first 12-month follow-up period (Table 1) For the remaining 92 patients, parasite isolates could only be taken at a single time point during our study either due to the absence of relapses or because taking additional bone marrow or splenic aspirates was not clinically justified. To identify if recurrent VL had been caused by re-expansion of persistent parasites within a patient after initial cure, we compared Nei’s genetic distances based on whole-genome SNP data between all pairwise sample combinations. Longitudinal samples of parasites from a patient were typically most closely related to parasite samples from the same patient at different times (Fig. 2A). For two time series samples (1023_EoT_1_primary_pos_0 and 1045_ToD_1_recurrent_pos_3), the genetically most similar samples came from another patient (Fig. 2A and S2A and B). For patient 1023, the genetic distance between samples gradually increased with time, while the first sample from this patient was very closely related to isolates from two other patients. However, for patient 1045, parasites isolated from the third VL episode were very different from parasites isolated 7 months later during the fourth recurrent VL episode, being more closely related to several isolates from other patients. This trend was confirmed when comparing sequential isolates from a patient to the initial isolate from the same patient (Fig. 2B). While within-host parasite changes gradually increased with time, those genetic distances were still very small compared to the median pairwise genetic distance in our entire data set. In this context, two isolates taken just before and after treatment of the fourth VL relapse for patient 1045 were clearly outliers, with a genetic distance similar to that of pairs of samples from different patients, indicating possible reinfection only in this case (Fig. 2B). Together, this suggests that the majority (6 of 7) of HIV-coinfected VL patients relapsed due to the original persistent parasite strain rather than reinfection with L. donovani.

**TABLE 1:** Summary of isolates from patients with multiple samples over time

Patient code	Disease/treatment/follow-up status^*a*^	HIV status	VL episode		Time from previous VL episode (mo)	Site of parasite isolation
			Category	Count		
1004	ToD	Pos	Recurrent	2	NA^*b*^	Spleen
1004	6-12m	Pos	Recurrent	3	13	Spleen
1023	EoT	Pos	Primary	0	NA	Bone marrow
1023	3m	Pos	Recurrent	1	5	Spleen
1023	6-12m	Pos	Recurrent	2	5	Spleen
1023	EoT	Pos	Recurrent	3	NA	Spleen
1026	ToD	Pos	Recurrent	5	NA	Spleen
1026	3m	Pos	Recurrent	6	4	No isolate
1026	6-12m	Pos	Recurrent	7	5	Spleen
1037	ToD	Pos	Recurrent	14		Spleen
1037	6-12m	Pos	Recurrent	15	9	Spleen
1040	ToD	Pos	Recurrent	10	NA	Spleen
1040	EoT	Pos	Recurrent	11	8	Spleen
1045	ToD	Pos	Recurrent	3	NA	Spleen
1045	6-12m	Pos	Recurrent	4	7	Spleen
1045	EoT-1	Pos	Recurrent	4	NA	Spleen
1078	ToD	Pos	Recurrent	11	NA	Spleen
1078	6-12m	Pos	Recurrent	12	7	Spleen

a ToD, time of diagnosis and study enrollment; EoT, end of treatment; 6-12m, recurrent VL during 6- to 12-month follow-up.

b NA, not available.

### Reduction in parasite heterozygosity during within-host evolution.

As persistent *Leishmania* populations seem to play a dominant role in recurrent VL in HIV-positive patients, we investigated whether particular patterns of genomic change might be associated with this prolonged within-host evolution. Ideally, we would compare longitudinal samples within the same patient over multiple relapses of VL, but the ethical constraints on taking appropriate isolates imply that relatively few such sample sets could be taken (see Table 1). The largest proportions of our parasite samples were isolated from different patients with primary VL and at different numbers of recurrent VL episodes. The identification of convergent genetic changes that could indicate functionally relevant adaptations to prolonged immune challenge is more difficult from these cross-sectional data, as changes will arise in different genomic backgrounds, and within-host adaptation will be conflated with genetic variation that was present in the initially infecting strain.

**FIG 3:**
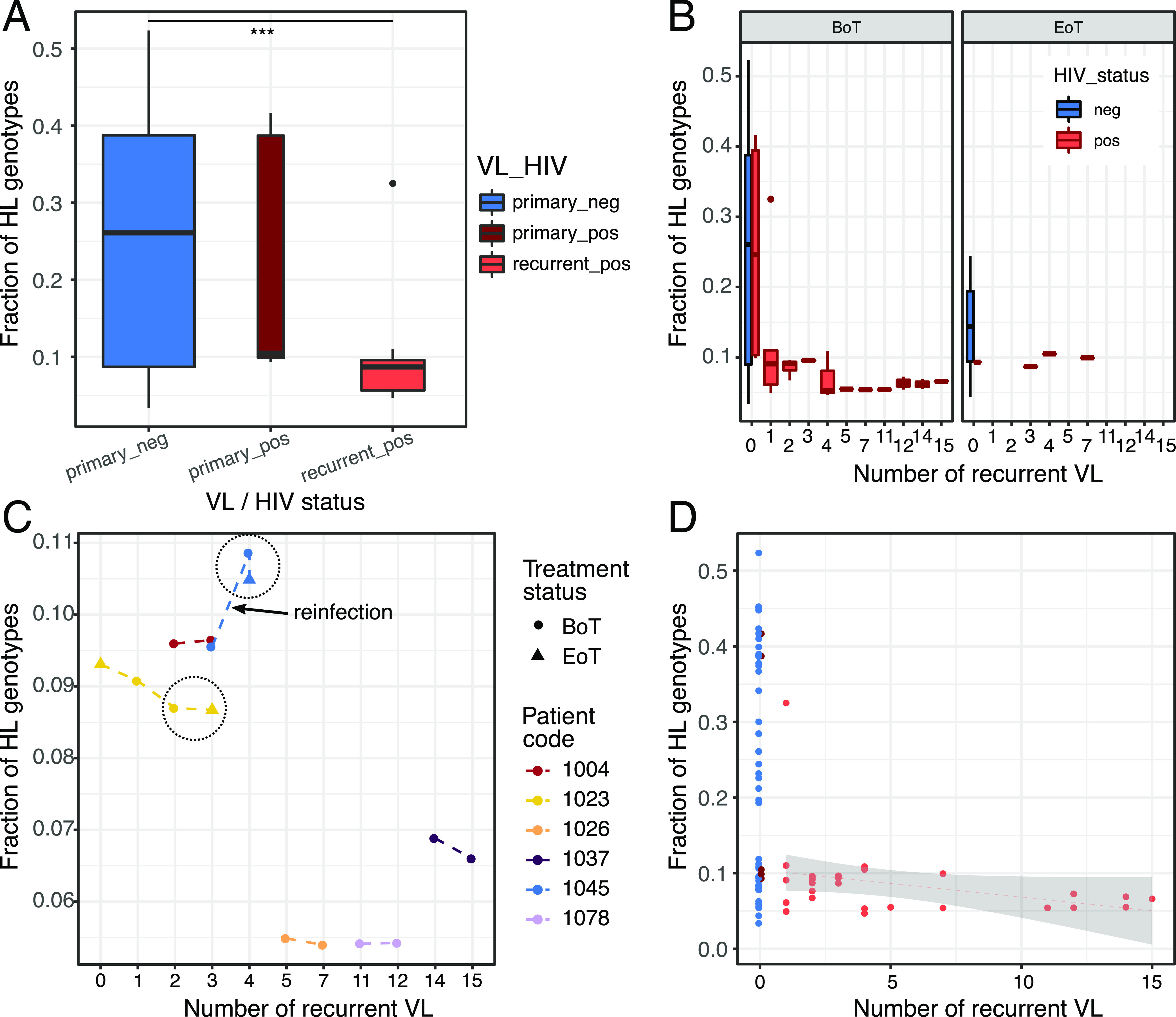
Parasite genomic changes with recurrent VL. (A) Heterozygosity (proportion of variable loci called heterozygous genotypes [HL]) in isolates from primary VL in HIV-negative patients (primary_neg), primary VL in HIV-infected patients (primary_pos), and relapse VL episodes in HIV-positive patients (recurrent_pos). There is a significant difference in heterozygosity between “primary_neg” and “recurrent_pos” (ANOVA, Tukey’s HSD; ***, FDR < 0.001). (B) Heterozygosity values (as shown in panel A) for each number of episodes of VL relapse for isolates taken before (BoT) and at the end (EoT) of treatment for that VL episode. (C) Fraction of heterozygous (HL) genotypes for samples from patients with multiple isolates taken. The large black circles group isolates from consecutive sampling events for a patient before and after treatment (dots indicate samples isolated before treatment [BoT] and triangles at the end of treatment [EoT]). The line segment connecting the two isolates suspected to be a reinfection event is indicated (see Fig. 2). (D) Fraction of heterozygous genotypes categorized by VL and HIV status shown against the number of recurrent VL across patients. A linear model fit is shown for isolates from the HIV-positive VL relapse cohort.

We thus first inspected more general patterns of genomic change with recurrent VL. The isolates represent samples of the diversity of parasites present in the infected tissue. From a previous analysis, it is clear that diploid SNP variants called by GATK can show allele frequencies far from the 0.5 expected for a diploid genotype (22). We therefore reasoned that the proportion of variable sites called as heterozygotes would be a good approximation of the genetic diversity of the infecting infrapopulation and give some indication of the effective population size. While we refer to this measure as “heterozygosity” it represents a combination of heterozygous differences within individual parasite clones and genetic differences within a diverse and potentially polyclonal infection. In our data, heterozygosity was much lower in isolates from recurrent VL in HIV-positive patients than in isolates from primary VL in HIV-negative patients (Fig. 3A) (analysis of variance [ANOVA], F = 16.25, *P* value = 8.52 × 10^−7^; Tukey’s HSD for recurrent_pos versus primary_neg, FDR = 0.0000004; recurrent_pos versus primary_pos, FDR = 0.0893283; primary_pos versus primary_neg, FDR = 0.8705747). Isolates from primary episodes of VL in HIV-infected patients had intermediate levels of heterozygosity, with a greater difference of the “primary_pos” cohort to the “recurrent_pos” than to the “primary_neg” cohort. This analysis left it unclear whether reduction in heterozygosity was associated with multiple episodes of VL disease only or if differences between the primary VL in HIV-positive and HIV-negative patients were also present. Therefore, we tested whether the fraction of heterozygous sites in primary VL isolates varied with HIV infection status when the reported length of illness before parasite isolation or the estimated parasite load at the time of isolation were taken into account. However, neither of these covariates had a significant effect on heterozygosity, and no difference between HIV categories was observed (see Fig. S3A and B).

10.1128/mBio.00971-21.2 FIG S2 Heat maps of Nei’s distances for isolates of patients 1023 and 1045. Download FIG S2, PDF file, 0.09 MB. Copyright © 2021 Franssen et al. 2021 Franssen et al. https://creativecommons.org/licenses/by/4.0/ This content is distributed under the terms of the Creative Commons Attribution 4.0 International license.

10.1128/mBio.00971-21.3 FIG S3 Relationship of the heterozygous fraction with other disease phenotypes. Download FIG S3, PDF file, 0.07 MB. Copyright © 2021 Franssen et al. 2021 Franssen et al. https://creativecommons.org/licenses/by/4.0/ This content is distributed under the terms of the Creative Commons Attribution 4.0 International license.

Next, we investigated the timing of the reduction in heterozygosity over the course of primary and relapse VL episodes. In the cross-sectional comparison, there was a strong difference in heterozygosity between isolates from patients with primary infections and those from patients experiencing the first relapse of VL (Fig. 3A), suggesting that treatment of primary VL itself already reduced heterozygosity levels. To test this directly, we looked at how heterozygosity of parasite isolates varied with the number of previous VL episodes and compared samples taken before and after treatment and in both HIV-positive and -negative patients (Fig. 3B). For the primary VL data, all three posttreatment samples suggested a noticeable reduction in heterozygosity levels compared to that in before-treatment isolates, but the low sample size impeded assessing statistical significance (Fig. 3B). Moreover, a small treatment effect was also still present for recurrent VL in our time series data (Fig. 3C, patients 1023 and 1045). While the reduction in heterozygosity between primary VL and the first relapse of VL was strongest, there was a trend for a linear decrease in heterozygosity of small effect size from the first VL relapse and subsequent recurrence of VL, though only significant at a lenient threshold of α = 0.1 (linear model fraction of heterozygous genotypes [fHL] ∼ number of relapses, *P* value = 0.0798) (Fig. 3D). This relationship was also reflected in our within-patient longitudinal data, where parasite heterozygosity levels decreased through time with additional relapse episodes. The only exception was a clear increase for patient 1045, for whom we had previously suggested reinfection (Fig. 3C). Taken together, these data suggest a strong effect of initial drug treatment on heterozygosity levels, followed by a weaker continuous effect, presumably due to repeated population size decreases during drug treatment.

### Infections in recurrent VL are as complex as primary infections.

The observed reduction of heterozygosity with recurrent VL could have been partly due to clonal diversity compared to a heterozygous allele shared across all cells in a population. Therefore, we investigated the amount of possible clonal diversity (the complexity of infection) in our isolates. By inspecting the distributions of minor allele frequencies at diploid chromosomes, we categorized them into isolates likely to come from single clonal infections (see Fig. S4A), isolates potentially representing complex infections with multiple clones (Fig. S4C), and those with slightly noisy allele frequency distributions that either represented very few variants or had a few variants present at unexpected frequencies (Fig. S4B and S5). Despite this somewhat subjective assessment, complex infections were clear outliers in terms of mean distance from the expected allele frequency and the variance of observed frequencies (Fig. S4D). Complex infections were found in 9% (6/68) of our primary isolates and 14% (4/29, one additional technical replicate) of the isolates from recurrent VL. None of our within-patient longitudinal data gained complexity with time, but one lost complexity between recurrent VL episode 1 versus 2 (patient 1040). The four complex isolates from recurrent VL were from episodes 1, 2, and twice from episode 10. Noisy profiles were more common in isolates from recurrent VL likely due to the small number of heterozygous sites in these isolates that reduced the signal-to-noise ratio. If the reduced heterozygosity we observe in recurrent VL was due to loss of clones in complex infections, we would expect complex infections to be common in primary isolates and less common in isolates from recurrent VL, which was not the case for our samples.

10.1128/mBio.00971-21.4 FIG S4 Use of allele frequency estimates to evaluate clonal diversity of individual isolates. Download FIG S4, PDF file, 0.3 MB. Copyright © 2021 Franssen et al. 2021 Franssen et al. https://creativecommons.org/licenses/by/4.0/ This content is distributed under the terms of the Creative Commons Attribution 4.0 International license.

10.1128/mBio.00971-21.5 FIG S5 Allele frequency profiles for isolates from patient 1045. Download FIG S5, PDF file, 0.2 MB. Copyright © 2021 Franssen et al. 2021 Franssen et al. https://creativecommons.org/licenses/by/4.0/ This content is distributed under the terms of the Creative Commons Attribution 4.0 International license.

### Unclear role of variation in chromosome dosage in within-host heterozygosity reduction.

**FIG 4:**
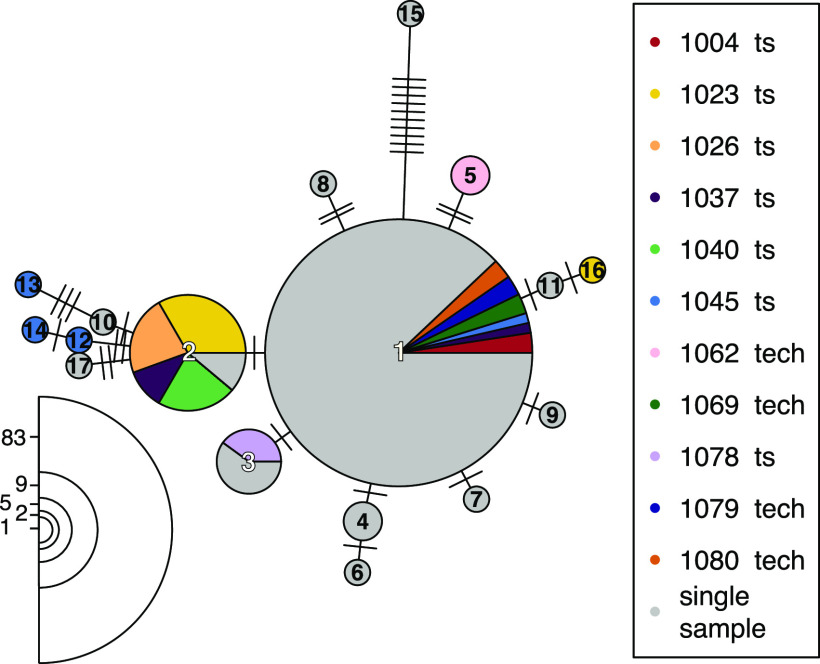
Network of aneuploidy profiles. Aneuploidy profiles of all 113 parasite isolates are shown in a minimum spanning network. Each circle represents a distinct aneuploidy profile, and edges between circles indicate similarity between profiles, with the number of edge ticks indicating the total somy difference between profiles. Colors indicate patient origin, with patients represented by only single isolates all gray and those with either multiple temporal samples (ts) or with aliquoted isolates (tech) in unique colors. Circle sizes represent the numbers of isolates showing a particular profile, and numbers for each circle size are indicated at the bottom left. Circles are numbered to identify each aneuploidy profile, and details of the somy patterns and abundance of each profile are listed in Table 2. The largest circle (profile 1) represents the diploid condition but with a tetrasomic chromosome 31.

*Leishmania* species are known to vary widely in chromosome copy number, with even different cells within a single clonal population varying in chromosome dosage (termed mosaic aneuploidy). This characteristic is generated through rapid change in chromosome copy number between mother and daughter cells for a subset of chromosomes, leading to cells having different aneuploidy profiles of chromosome dosage. Reductions in copy number are expected to reduce heterozygosity of individual parasites (26). Moreover, frequent turnover of chromosome copies should lead to a reduction in observed strain allelic diversity if a particular haplotype is favored by selection or drift is high due to small population sizes. While previous work suggests that aneuploidy mosaicism and turnover are rare in the mammalian host compared to that in promastigote culture (27), it could still contribute to the observed reduction of heterozygosity during within-host evolution. As we only sequenced DNA extracted from pools of cells for each parasite isolate instead of from individual cells, we cannot observe cell-to-cell variation. Therefore, we compared aneuploidy profiles between isolates from our time series data as well as for independently sequenced aliquots from some isolates as a conservative estimate of the degree of aneuploidy variation in our study (see Table S2). The aneuploidy level was generally low in our sample cohort, with 73.5% (83/113) of all samples having a population aneuploidy profile of diploid chromosomes apart from a tetraploid chromosome 31 (see Fig. S6). Time series samples and replicate primary isolates were from a total of 11 patients, and 72.7% (8/11) of those patients with multiple samples had the same population-wide aneuploidy profiles in all samples. In contrast, isolates from patients 1023, 1037, and 1045 showed some aneuploidy variation either between sampling events or between aliquots from the same biopsy sample (Fig. 4; Tables 2 and S2). This suggests that a low level of aneuploidy variation is present within patients, though we cannot definitively associate this variation with the reduction in heterozygosity we observed in isolates from patients with repeated relapses.

**TABLE 2:**
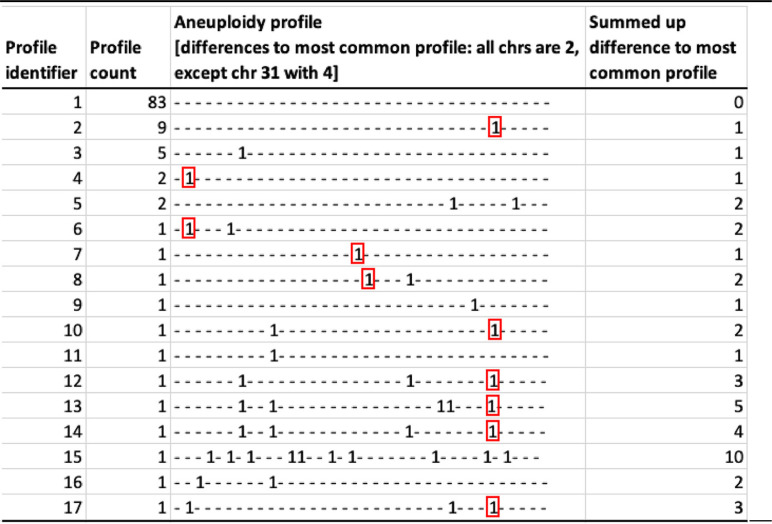
Summary of aneuploidy profiles and their abundances across all parasite isolates

a Negative differences to the dominant aneuploidy profile are indicated in red.

10.1128/mBio.00971-21.6 FIG S6 Aneuploidy profiles of all 113 parasite samples. Download FIG S6, PDF file, 0.1 MB. Copyright © 2021 Franssen et al. 2021 Franssen et al. https://creativecommons.org/licenses/by/4.0/ This content is distributed under the terms of the Creative Commons Attribution 4.0 International license.

10.1128/mBio.00971-21.10 TABLE S2 Aneuploidy profiles and metadata summary of all isolates from patients with time series data and/or replicates of primary isolates. Download Table S2, PDF file, 0.7 MB. Copyright © 2021 Franssen et al. 2021 Franssen et al. https://creativecommons.org/licenses/by/4.0/ This content is distributed under the terms of the Creative Commons Attribution 4.0 International license.

### No signal of drug resistance evolution during repeated treatment of recurrent VL.

**FIG 5:**
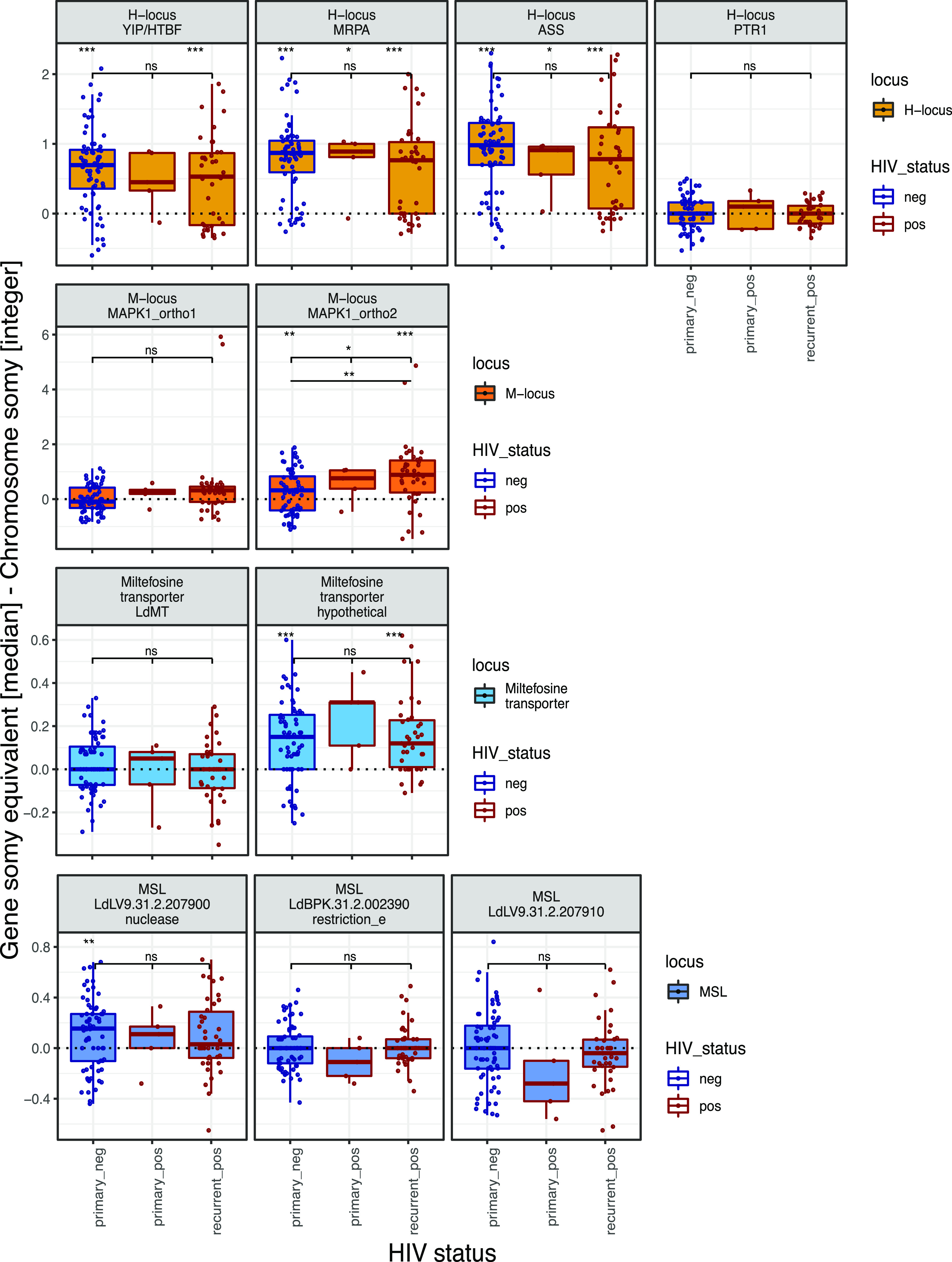
Gene copy numbers at known drug resistance loci across disease cohorts. Gene copy numbers at four drug resistance loci are shown for the three disease cohorts: “primary_neg,” “primary_pos,” and “recurrent_pos.” Gene copy numbers are estimated as differences in somy equivalent to the respective chromosome somy. Top row significance levels in each plot indicate FDR values from three one-sample *t* tests; in the second row, ANOVA results are shown, and in the third row, Tukey’s HSD (if applicable) results are shown. ***, *P* < 0.001; **, *P* < 0.01; *, *P* < 0.05; ns, not significant.

A possible explanation for the survival of parasites through treatment is the evolution of drug resistance within the parasite infrapopulation, which would also contribute to the high rate of treatment failure during subsequent VL relapses. However, so far, there is little direct evidence that treatment failure in VL/HIV patients is largely driven by drug resistance. We investigated whether known markers for drug resistance in *Leishmania* were present in our study cohorts and compared their frequencies between primary and recurrent VL. Gene copy numbers were estimated for genes within four loci previously suggested to be involved in drug resistance, including the H locus (28), the M locus (MAPK1 gene [29]), both associated with resistance to antimonial drugs, the miltefosine transporter (30), and the miltefosine sensitivity locus (MSL) (31). We tested for change in copy number at each of these four loci in HIV-negative primary VL cases (“primary_neg”), HIV-positive primary cases (“primary_pos”), and from relapse cases in HIV positive patients (“recurrent_pos”). Several genes showed increases with respect to the baseline chromosome copy number for the two larger groups (primary_neg and recurrent_pos), including YIP, MRPA, and ASS at the H locus and at a gene associated with the miltefosine transporter (annotated as a hypothetical protein in the LV9 reference genome; one sample *t* tests, FDR < 0.001) (Fig. 5); only in two of these above cases the primary_pos cohort had a significant increase in copy number compared to the chromosome background, but this cohort also had far fewer samples, making it hard to detect small effect size changes (one sample *t* tests, FDR < 0.05) (Fig. 5). Comparison between all three groups for each gene did not show any differences between the three disease categories except for the second MAPK1 ortholog (ANOVA, FDR < 0.05) (Fig. 5). The increased dosage at the H locus in our cohort is in line with previous findings from Ethiopian isolates (22), but as both this increase and the dosage increase of the miltefosine transporter-associated gene are similarly present in both HIV-positive and -negative patients, they cannot explain VL relapse. Only for the second ortholog of the MAPK1 gene does our data support a copy number increase with relapse compared to primary VL, which could indicate evolution of drug resistance during repeated treatment (Fig. 5). This comparison was, however, only significant at an α of 0.05 and was not observed in longitudinal comparisons within a patient (see Fig. S7). A larger sample size would be needed to support the role of this variant as an important factor in driving worse treatment outcomes in VL/HIV patients.

10.1128/mBio.00971-21.7 FIG S7 Gene copy numbers at known drug resistance loci for time series isolates. Download FIG S7, PDF file, 0.1 MB. Copyright © 2021 Franssen et al. 2021 Franssen et al. https://creativecommons.org/licenses/by/4.0/ This content is distributed under the terms of the Creative Commons Attribution 4.0 International license.

It has previously been suggested that different aneuploidy patterns themselves can be adaptive in new environments (27, 32). We noticed that the dominant aneuploidy profile described above (all chromosomes are diploid except for a tetraploid chromosome 31) was more frequent in primary VL isolates before treatment (88.55% [62/70]) than in isolates from recurrent VL (44.7% [17/38]) (see Fig. S8A). This poses the question of whether these different aneuploidy patterns might be adaptive in HIV patients, allowing parasites with these patterns to become more frequent in these patients during repeated VL episodes. We therefore tested if divergence from the dominant aneuploidy profile increased with the number of episodes of VL relapse (Fig. S8C and D), but there was only support at an α of 0.1 for this trend (linear model, sum of the difference profile ≈ number of relapses, *P* value = 0.0561). In total, 13 of 25 isolates from different patients with recurrent VL showed six different divergent aneuploidy profiles (Fig. S8B). In those profiles, a total of six chromosomes showed changes in somy in a few combinations. Four of the deviations were present in isolates from different patients, with the most frequent changes occurring in 28% and 20% of all isolates from recurrent VL showing a reduction from a somy of 4 to 3 for chromosome 31 and a somy increase from 2 to 3 for chromosome 7, respectively (Fig. S8B). While within-patient environments are expected to be heterogeneous due to both patient-specific differences and different drug treatment histories, this convergence might suggest an adaptive benefit of reduced chromosome 31 dosage or other convergent somy increases during prolonged parasite evolution in HIV patients with recurrent VL.

10.1128/mBio.00971-21.8 FIG S8 Aneuploidy changes of parasites from patients with recurrent VL. Download FIG S8, PDF file, 0.1 MB. Copyright © 2021 Franssen et al. 2021 Franssen et al. https://creativecommons.org/licenses/by/4.0/ This content is distributed under the terms of the Creative Commons Attribution 4.0 International license.

## DISCUSSION

We have shown that, in northern Ethiopia, the same diverse population of parasites is responsible for VL and VL/HIV cases. Infection of HIV-positive patients and the associated clinical manifestations (25) are thus not due to phylogenetically distinct strains. However, we observe a small degree of genetic structure between isolates from VL and VL/HIV patients with borderline statistical significance. While we cannot exclude the possibility that specific groups of strains may have a higher propensity to infect HIV-positive patients, we propose that this is likely to be due to geographic structure in the parasite population that is correlated with geographic differences in HIV prevalence. Many of the VL patients in this region of Ethiopia (8), including 84.8% of the patients in this cohort (25), are migrant workers who move from regions of nonendemicity for seasonal agricultural work. Possibly due to their origin from a region of nonendemicity and the working and living conditions on the farms, they are frequently infected with *Leishmania* and develop clinical VL. HIV infection is also more frequent in the migrant worker population (33), introducing an association between the location of infection and HIV status. Identifying the actual location these patients acquired VL infections would require a detailed study based on these farms. These are extremely challenging environments to work in, because of both the lack of basic infrastructure and the currently poor security situation in the farming area close to the border with Sudan.

Our within-patient longitudinal data suggest that recurrent VL in HIV-coinfected patients is predominantly caused by the re-expansion of persistent parasites. In six of seven patients for which we obtained longitudinal samples from subsequent relapses of VL, parasites from the same patient were clearly most closely related to each other. For one patient (1023), parasite isolates were extracted at four different time points and showed a gradual divergence from the initial isolate, as expected if parasites from the originally infecting population are responsible for relapses. In only a single case (patient 1045), isolates taken at the third and fourth episodes of VL for this patient (the time of initial diagnosis in this study and 7 months later) were as divergent as isolates from different patients. We interpret this as likely due to reinfection of this patient following cure. We cannot rule out the possibility that this patient was initially infected with multiple parasite strains and, by chance, different strains from an initial infection were observed in subsequent isolates. However, we think this scenario is unlikely, as we cannot see any strong indication that any isolates from this patient are complex infections (see Fig. S5 in the supplemental material). We conclude that, in this single case, reinfection was contributing to the relapse of VL. The observation in the other six cases that parasite isolates from a single patient gradually diverge with the number of VL relapses provides the first genetic evidence that successful treatment of VL—at least in HIV-coinfected patients—is not sterile and is responsible for relapse.

It is clear from animal models of cutaneous leishmaniasis that *Leishmania* can enter a distinct, quiescent nonreplicating state (34) that has been associated with persistence in tissues. Parasite persistence certainly also occurs clinically. Parasite DNA and live parasites have been isolated from healed cutaneous leishmaniasis (CL) scars (35) and from the skin of cured post-kala-azar dermal leishmaniasis (PKDL) patients (36). Reappearance of active CL has been recorded up to 50 years after patients leave an area of endemicity (37). This phenomenon also occurs with VL (38), sometimes with fatal consequences (39). The presence of persistent parasites may be vital in establishing effective long-term immunity to leishmania reinfection (40). The role of persistent parasites is also supported by our finding of much lower heterozygosity in parasite isolates from relapse episodes of VL, with a gradual reduction in heterozygosity with multiple relapses. Together, it suggests that heterozygosity in the infrapopulation of parasites within a host is lost due to strong population size reduction during initial VL treatment. In many HIV-positive patients, the population re-expands, causing relapse of disease symptoms: treatment of recurrent episodes of VL then leads to repeated population bottlenecks and continuing small reductions in heterozygosity. While this scenario is consistent with our data, a number of questions remain. We find a number of isolates that show unusual allele frequency distributions suggestive of complex infections caused by multiple clones; these were no more frequent in primary isolates than those taken during relapse episodes of VL, suggesting that loss of clonal complexity in the infections is not responsible for the observed loss of heterozygosity. We note that this is based on only a single isolate from spleen or bone marrow per patient at any timepoint, and it is possible that parasite populations are present in other host tissues such as the skin (41).

We find complex infections present as late as the tenth episode of VL relapse, which is very puzzling; although for these isolates, the total heterozygosity is low enough that it might have been generated during within-host evolution. The origins of the heterozygosity present in the initial infection is not completely clear. One scenario could be the frequent presence of multiple infecting clones in the initial sandfly bite, most of which are lost on initial treatment. This would imply either that flies frequently pick up multiclonal infections from single mammal hosts or that they frequently bite multiple infected hosts before transmitting an infection. A few studies have either investigated multiple isolates collected from individual hosts (42, 43) or directly investigated the diversity of parasites in tissues (44) or vectors (45, 46) and find some evidence for polyclonal infections, but such reports seem to be rare. Similarly, individual sand flies biting more than two infected hosts seems unlikely to be very frequent when prevalence of *Leishmania* infections is not very high. However, it might be promoted if prevalence is much higher in very focal areas and because multiple blood meals appear to greatly promote *Leishmania* infections in the sandfly gut (47). This could tend to increase the number of flies transmitting infections picked up from multiple hosts. In general, we know too little about the life span or biting rates of sandflies to evaluate this possibility, even in the best studied foci of VL (48). Prevalence in this region is not well understood, as quantitative data are either old (e.g., see reference 49) or restricted to subpopulations such as HIV patients (e.g., see reference 50) or patients reporting with suspected VL (e.g., see reference 51), although detailed information about one small neighboring focus is available (52).

While, to our knowledge, we present the first longitudinal data from cases of *Leishmania* relapse, a number of studies have used genomic approaches to investigate recurrent malaria infections, which have demonstrated that relapse is due to parasite recrudescence (53–55). While these studies do not identify any genetic variation associated with relapse, they confirm that this is largely due to regrowth of one, or few, clonal lineages from initially complex infections (54, 55). One key limitation of our analysis of longitudinal changes in parasite populations is the ethical constraint on the availability of parasite isolates, which we could only obtain when invasive spleen or bone marrow aspirates were taken by clinicians for diagnostic purposes. Isolating parasites from blood samples would be less invasive (56, 57), but these approaches are little used and require conditions not generally available in resource-limited clinical settings. Culture-free approaches could be feasible (44), although parasitemia in many VL patients is likely to be too low for current approaches (e.g., see reference 58).

The heterozygosity we report in primary VL isolates appears to be due to both complex infections and genuine heterozygous variants within a parasite clone. These multiple causes of heterozygosity are one reason we have not attempted to use our diversity estimates to directly quantify the size of any population bottleneck in these patients. Another barrier to quantification is the rather complex genetics of *Leishmania*. *Leishmania* can reproduce clonally but can also have normal meiotic sex (59), which is probably rare (60), and might also be able to reproduce parasexually (61). *Leishmania* populations also show extensive variation in aneuploidy (22, 42, 61–65), but it is unclear to what extent this occurs in the field (27, 44). Indeed, this aneuploidy variation seems to occur particularly rapidly in culture (26, 61). Very rapid turnover of chromosome copies should decrease heterozygosity within individual cells, but it is not clear that it should impact heterozygosity measured at the level of an entire population of cells (61). Robust approaches to estimate parameters such as the change in population size upon drug treatment from patterns of genetic variation between isolates or through time in a longitudinal sample would need to take these unusual features of *Leishmania* genetics into account.

We report a relatively small amount of aneuploidy variation between isolates, which could contribute to the loss of heterozygosity we observed with treatment of VL relapse. An important caveat to these results is that we estimate ploidy from DNA extracted from the entire population of cells present in the primary sample taken to isolate parasites. Using these initial cultures minimizes the possibility that aneuploidy changes occur as the parasites adapt to *in vitro* growth, but there could still be some difference to the aneuploidy profile of parasites in the patients themselves (27, 44). In addition, the measure will be conservative in identifying variation in somy, as it ignores variation between parasite cells within a single isolate. Directly demonstrating that aneuploidy turnover occurs and quantifying its impact on heterozygosity differences between isolates would require either multiple samples from a much larger set of patients or investigating the genomes of individual cells directly, which is now possible but remains costly and technically challenging and gives much less clear results than sequencing larger amounts of material (66).

The parasites we investigate from recurrent VL cases have not been phenotypically investigated for drug sensitivity or resistance, and we do not generally identify changes at drug resistance loci in the genomic data from these parasites. We thus presume that drug resistance is not responsible for the increased difficulty in treating patients with repeated relapses of VL (18, 25) and the failure to successfully treat these patients long term. Indeed, it is unclear in general to what extent treatment failure in *Leishmania* is due to drug resistance (67–69). However, we can only screen for known genetic variants associated with resistance, and our knowledge of the genetic basis of drug resistance in *Leishmania* is very incomplete (68) and largely based on studying parasite clones selected for resistance in the laboratory (70). In particular, the markers that are available for VL have largely been identified in Indian L. donovani or in L. infantum. African L. donovani populations are genetically highly differentiated from populations elsewhere in the world (22), and it is possible that the genetic basis of drug resistance in Africa, if any, could be very different. In particular, many known markers are for resistance to miltefosine, which is not widely available in Africa (71). Little work has been done to investigate drug resistance markers in African visceral leishmaniasis, where drug resistance is not thought to be a significant clinical problem (72), and no genetic markers have—to our knowledge—been identified in the region. Identifying such resistance in recurrent VL/HIV would be complicated by the complex history of drug exposure for many of these patients, as clinicians frequently resort to second-line drugs or combinations in an attempt to improve treatment outcomes (16, 18, 73).

### Conclusion.

We show that a diverse population of L. donovani present in northern Ethiopia is responsible for visceral leishmaniasis in both single VL infections and in VL/HIV coinfections, with no clear genome-wide genetic differentiation between parasites isolated from the two patient groups. Our data suggest that most relapses are caused by recrudescence (regrowth) of the initial infecting parasite population rather than reinfection from a subsequent infected sandfly bite. Parasite populations present in primary VL episodes are more diverse than those isolated during VL relapses and that the infrapopulation of parasites within a host patient loses genetic diversity with increasing numbers of subsequent relapses. This loss of diversity is presumably due to population bottlenecks induced by treatment of each episode of disease. We observed complex multiclonal infections, but these were not more common in primary infections. We propose that infecting parasites are able to reestablish despite clinical cure after antileishmanial treatment, due to the dysfunction of the immune system of patients coinfected with VL and HIV (25) that is unable to prevent re-expansion of the parasite infrapopulation leading to VL relapse. This implies that better approaches for identifying patients at highest risk of relapse (25) and alternative treatment that allows these patients to restore functioning immunity might help prevent subsequent relapses and so improve clinical outcomes for VL/HIV patients.

## MATERIALS AND METHODS

This study was approved by the Institutional Review Board (IRB) of the University of Gondar (reference O/V/P/RCS/05/1572/2017), the National Research Ethics Review Committee (NRERC; reference 310/130/2018), and Imperial College Research Ethics Committee (ICREC; 17SM480). Informed written consent was obtained from each patient. The patients were all recruited at the time of VL diagnosis (ToD) at the Leishmaniasis Research and Treatment Center in Gondar, Amhara National Regional State, Ethiopia, between December 2017 and May 2019 and were each followed up for 3 years (25).

### Parasite isolation and culturing.

Isolates were obtained from splenic or bone marrow aspirates from VL and VL/HIV patients. Splenic aspiration can only be performed if the spleen is palpable at least 3 cm below the costal margin, there are no signs of bleeding, jaundice, or severe anemia and platelet count of <40,000/ml. Otherwise, bone marrow aspiration was performed. Immediately after collection, tissue aspirations were cultured as previously described (74) and kept in primary medium until DNA was extracted using DNeasy Blood & Tissue extraction kits (Qiagen, Hilden, Germany) according to the manufacturer’s instructions. Extracted DNA samples were stored at −20°C before library preparation and sequencing.

### Sequencing.

Genomic DNA was sheared into 400- to 600-bp fragments by focused ultrasonication (Covaris Inc.), and Illumina libraries were prepared using an NEB Ultra II custom kit (75) and then cleaned up using Agencourt AMPure XP SPRI beads (Beckman Coulter). The resulting libraries were sequenced as multiplexed pools of 96 samples and 17 samples (combined with samples from unrelated studies) as 151-bp paired-end reads on the Illumina HiSeq X10 platform.

### Genomic analysis pipeline.

Sequencing data for all samples were trimmed with Trimmomatic version 0.39 (76) to remove putative remains of adapter sequences and trim low-quality 3′ end using parameters “ILLUMINACLIP:PE_adaptors.fa:2:30:10 TRAILING:15 SLIDINGWINDOW:4:15 MINLEN:50” in paired-end mode. Resulting paired-end reads were mapped with BWA version 0.7.17 (77) using the bwa mem -M option against the reference genome of the L. donovani isolate LV9 from Ethiopia, version 43 (https://tritrypdb.org/). SNPs were called on the resulting individual bam files using GATK version 4.1.2.0 (78, 79) with the Haplotype caller and parameters “-ERC GVCF –annotate-with-num-discovered-alleles –sample-ploidy 2” to generate gvcf files for each sample. “GenomicsDBImport” and “GenotypeGVCFs” were used to combine individual gvcf files and call SNPs for the sample cohort. SNPs were then hard filtered with “VariantFiltration” using the following filters: QD, <2.0; MQ, <50.0; FS, >20.0; SOR, >2.5; BaseQRankSum, <−3.1; ClippingRankSum, <−3.1; MQRankSum, <−3.1; ReadPosRankSum, <−3.1; and DP, <6. For subsequent phylogenetic and population genetic analysis, SNPs with more than 20% missing calls across samples were removed, reducing the total number of SNPs to 370,714.

### Phylogenetic reconstruction and phenotype association.

Phylogenetic trees were reconstructed using a distance-based method: pairwise distances between samples were calculated as Nei’s genetic distances using the R package StAMPP version 1.6.1 (80), and trees were reconstructed with neighbor joining using the R package ape version 5.4 (81). To obtain bootstrap values, the total number of SNPs was drawn from all SNPs with replacement for each of 100 bootstrap replicates. For each, Nei’s distance matrices were calculated and individual replicate trees reconstructed with neighbor joining. Reported values at the nodes of the original tree are percentages of the respective node across the replicate trees. Associations of HIV and VL recurrence status with the phylogeny were tested based on the pairwise genetic distance matrix (Nei’s D) using ANOSIM analysis (R package vegan, version 2.5-6) and Blomberg’s *K* (R package phytools, version 0.7-47) (82).

### Population genomics analysis.

Population genomic analysis was based on genotype calls for each sample for the previously described 370,714 SNP sites. All analysis and plotting were performed in R (83). Alleles at all SNP sites were polarized based on their frequencies across all primary isolates and encoded as either H or L for “high” or “low” frequency, respectively, across all samples isolated from patients with primary VL. During time series analysis, it was noticed that the two samples isolated from patient 1040 had unusually high fractions of LL genotypes during recurrent VL that were much higher than in isolates from other patients with recurrent disease. We therefore checked if this could be a biological signal or rather a technical artifact. Generally, high fractions of LL genotypes in a sample were associated with higher missingness of genotype calls, suggesting that an extreme fraction of LL genotypes might rather be a genotype calling artifact in samples with less power for accurate SNP calling. We therefore excluded 8 samples with a fraction of ≥0.002 unknown genotype calls from this analysis (1002_ToD_1_primary_neg, 1025_ToD_1_primary_neg, 1040_EoT_2_recurrent_pos, 1040_ToD_1_recurrent_pos, 1041_ToD_1_primary_neg, 1061_ToD_1_primary_neg, 1077_ToD_1_recurrent_pos, and 1079_ToD_1_recurrent_pos).

### Evaluation of complexity of infection.

In a strictly clonal population, allele frequency distributions have a clear expectation given the ploidy of the respective chromosome; e.g., for a diploid chromosome, the allele frequency distribution should peak at 0.5, and for a triploid chromosome, it should peak at one-third and/or two-thirds. We used this expectation to evaluate whether each isolate was most likely to represent (i) a single clonal infection where the allele frequencies were as expected, (ii) noisy profiles with very few variants or without very clear peaks, or (iii) profiles with peaks at unexpected allele frequencies, likely to represent complex infections with a mixture of clones (see Fig. S4A to C in the supplemental material). For each isolate, the allele frequency distribution for all variants called in that isolate on each diploid chromosome and the absolute difference between each allele frequency and the expected frequency of 0.5 were calculated. The distribution of these differences was summarized by its mean and standard deviation (Fig. S4D and E), confirming that complex infections were distinct from either single infection profiles or those with little or noisy signal.

### Aneuploidy and copy number variant analysis.

For copy number estimation, previously described bam files were further processed to estimate somies of individual samples and chromosomes as well as the relative copy number of genes previously suggested to be involved in drug resistance. GATK version 3.6 was used to perform indel realignment using “RealignerTargetCreator” to identify and “IndelRealigner” for realignment in the identified regions (78). Then bam files were filtered for proper pair mapping, and duplicates were removed using SAMtools version 1.9 with the parameters “-F 1024 -f 0 × 0002 -F 0 × 0004 -F 0 × 0008.” Genome coverages were estimated with bedtools genomecov version 2.29 and parameters “-d -split.” Data were subsequently processed in R. Aneuploidy profiles for each sample were estimated using median chromosome coverage. The median chromosome coverage for each sample was used to estimate the chromosome-specific coverage, and across all chromosomes of a sample, the median value was assumed to present the diploid stage. So for each sample, chromosome-specific somy equals the chromosome-specific coverage divided by the median chromosome-specific coverage across all chromosomes times 2. Visualizations of aneuploidy profiles were performed in R with the package heatmap.2, and minimum spanning networks were visualized with the mst function of the pegas package (version 0.13).

To assess the coverage at genes of putative interest for drug resistance, coverage was estimated through the median coverage across bases in the respective gene. This coverage was translated into a somy equivalent by dividing the base coverage at each genomic position by the median chromosome coverage and multiplying with the chromosome-specific rounded somy. The somy equivalent change in coverage was estimated for genes from four loci previously associated with drug resistance, including the H locus, the M locus (MAPK1) associated with resistance to antimonial drugs, the miltefosine transporter and associated genes (84, 85), and the miltefosine sensitivity locus. Genes in the LV9 reference genome (version 43) were identified via ortholog annotation in TriTryp (https://tritrypdb.org/) of the respective gene names of L. infantum JPCM5 (version 41) as previously described (22); locus identifiers (IDs) for the LV9 genome annotation are included in Fig. 5.

### Data availability.

Sequencing reads from this project are all available from the European Nucleotide Archive and BioProject under accession number PRJEB30077.

## References

[1] Alvar J, Velez ID, Bern C, Herrero M, Desjeux P, Cano J, Jannin J, den Boer M, Who Leishmaniasis Control Team. 2012. Leishmaniasis worldwide and global estimates of its incidence. PLoS One 7:e35671. doi:10.1371/journal.pone.0035671. 22693548 PMC3365071

[2] Mathers CD, Ezzati M, Lopez AD. 2007. Measuring the burden of neglected tropical diseases: the global burden of disease framework. PLoS Negl Trop Dis 1:e114. doi:10.1371/journal.pntd.0000114. 18060077 PMC2100367

[3] Chappuis F, Sundar S, Hailu A, Ghalib H, Rijal S, Peeling RW, Alvar J, Boelaert M. 2007. Visceral leishmaniasis: what are the needs for diagnosis, treatment and control? Nat Rev Microbiol 5:873–882. doi:10.1038/nrmicro1748. 17938629

[4] Singh OP, Hasker E, Sacks D, Boelaert M, Sundar S. 2014. Asymptomatic *Leishmania* infection: a new challenge for *Leishmania* control. Clin Infect Dis 58:1424–1429. doi:10.1093/cid/ciu102. 24585564 PMC4001287

[5] McCall LI, Zhang WW, Matlashewski G. 2013. Determinants for the development of visceral leishmaniasis disease. PLoS Pathog 9:e1003053. doi:10.1371/journal.ppat.1003053. 23300451 PMC3536654

[6] Rijal S, Sundar S, Mondal D, Das P, Alvar J, Boelaert M. 2019. Eliminating visceral leishmaniasis in South Asia: the road ahead. BMJ 364:k5224. doi:10.1136/bmj.k5224. 30670453 PMC6340338

[7] Leta S, Dao TH, Mesele F, Alemayehu G. 2014. Visceral leishmaniasis in Ethiopia: an evolving disease. PLoS Negl Trop Dis 8:e3131. doi:10.1371/journal.pntd.0003131. 25188253 PMC4154678

[8] Gadisa E, Tsegaw T, Abera A, Elnaiem DE, den Boer M, Aseffa A, Jorge A. 2015. Eco-epidemiology of visceral leishmaniasis in Ethiopia. Parasit Vectors 8:381. doi:10.1186/s13071-015-0987-y. 26187584 PMC4506599

[9] Gelanew T, Cruz I, Kuhls K, Alvar J, Canavate C, Hailu A, Schonian G. 2011. Multilocus microsatellite typing revealed high genetic variability of *Leishmania donovani* strains isolated during and after a Kala-azar epidemic in Libo Kemkem district, northwest Ethiopia. Microbes Infect 13:595–601. doi:10.1016/j.micinf.2011.02.003. 21382503

[10] Alvar J, Bashaye S, Argaw D, Cruz I, Aparicio P, Kassa A, Orfanos G, Parreno F, Babaniyi O, Gudeta N, Canavate C, Bern C. 2007. Kala-azar outbreak in Libo Kemkem, Ethiopia: epidemiologic and parasitologic assessment. Am J Trop Med Hyg 77:275–282. doi:10.4269/ajtmh.2007.77.275. 17690399

[11] Lindoso JAL, Moreira CHV, Cunha MA, Queiroz IT. 2018. Visceral leishmaniasis and HIV coinfection: current perspectives. HIV AIDS (Auckl) 10:193–201. doi:10.2147/HIV.S143929. 30410407 PMC6197215

[12] Mohebali M, Yimam Y. 2020. Prevalence estimates of human immunodeficiency virus (HIV) infection among visceral leishmaniasis infected people in Northwest Ethiopia: a systematic review and meta-analysis. BMC Infect Dis 20:214. doi:10.1186/s12879-020-4935-x. 32164607 PMC7069024

[13] Albuquerque L. C P d, Mendonça IR, Cardoso PN, Baldaçara LR, Borges MRMM, Borges J.dC, Pranchevicius M. C d S. 2014. HIV/AIDS-related visceral leishmaniasis: a clinical and epidemiological description of visceral leishmaniasis in northern Brazil. Rev Soc Bras Med Trop 47:38–46. doi:10.1590/0037-8682-0180-2013. 24603735

[14] Hurissa Z, Gebre-Silassie S, Hailu W, Tefera T, Lalloo DG, Cuevas LE, Hailu A. 2010. Clinical characteristics and treatment outcome of patients with visceral leishmaniasis and HIV co-infection in northwest Ethiopia. Trop Med Int Health 15:848–855. doi:10.1111/j.1365-3156.2010.02550.x. 20487426

[15] Alemayehu M, Wubshet M, Mesfin N. 2016. Magnitude of visceral leishmaniasis and poor treatment outcome among HIV patients: meta-analysis and systematic review. HIV AIDS (Auckl) 8:75–81. doi:10.2147/HIV.S96883. 27042142 PMC4809333

[16] Diro E, Edwards T, Ritmeijer K, Fikre H, Abongomera C, Kibret A, Bardonneau C, Soipei P, Mutinda B, Omollo R, van Griensven J, Zijlstra EE, Wasunna M, Alves F, Alvar J, Hailu A, Alexander N, Blesson S. 2019. Long term outcomes and prognostics of visceral leishmaniasis in HIV infected patients with use of pentamidine as secondary prophylaxis based on CD4 level: a prospective cohort study in Ethiopia. PLoS Negl Trop Dis 13:e0007132. doi:10.1371/journal.pntd.0007132. 30789910 PMC6400407

[17] Diro E, Lynen L, Mohammed R, Boelaert M, Hailu A, van Griensven J. 2014. High parasitological failure rate of visceral leishmaniasis to sodium stibogluconate among HIV co-infected adults in Ethiopia. PLoS Negl Trop Dis 8:e2875. doi:10.1371/journal.pntd.0002875. 24854196 PMC4031116

[18] Mohammed R, Fikre H, Schuster A, Mekonnen T, van Griensven J, Diro E. 2020. Multiple relapses of visceral leishmaniasis in HIV co-infected patients: a case series from Ethiopia. Curr Ther Res Clin Exp 92:100583. doi:10.1016/j.curtheres.2020.100583. 32382359 PMC7198908

[19] Gelanew T, Kuhls K, Hurissa Z, Weldegebreal T, Hailu W, Kassahun A, Abebe T, Hailu A, Schonian G. 2010. Inference of population structure of *Leishmania donovani* strains isolated from different Ethiopian visceral leishmaniasis endemic areas. PLoS Negl Trop Dis 4:e889. doi:10.1371/journal.pntd.0000889. 21103373 PMC2982834

[20] Gebre-Michael T, Lane RP. 1996. The roles of *Phlebotomus martini* and *P. celiae* (Diptera: Phlebotominae) as vectors of visceral leishmaniasis in the Aba Roba focus, southern Ethiopia. Med Vet Entomol 10:53–62. doi:10.1111/j.1365-2915.1996.tb00082.x. 8834743

[21] Gebre-Michael T, Balkew M, Berhe N, Hailu A, Mekonnen Y. 2010. Further studies on the phlebotomine sandflies of the kala-azar endemic lowlands of Humera-Metema (north-west Ethiopia) with observations on their natural blood meal sources. Parasit Vectors 3:6. doi:10.1186/1756-3305-3-6. 20181077 PMC2829606

[22] Franssen SU, Durrant C, Stark O, Moser B, Downing T, Imamura H, Dujardin JC, Sanders MJ, Mauricio I, Miles MA, Schnur LF, Jaffe CL, Nasereddin A, Schallig H, Yeo M, Bhattacharyya T, Alam MZ, Berriman M, Wirth T, Schonian G, Cotton JA. 2020. Global genome diversity of the Leishmania donovani complex. Elife 9:e51243. doi:10.7554/eLife.51243. 32209228 PMC7105377

[23] Cotton JA, Durrant C, Franssen SU, Gelanew T, Hailu A, Mateus D, Sanders MJ, Berriman M, Volf P, Miles MA, Yeo M. 2020. Genomic analysis of natural intra-specific hybrids among Ethiopian isolates of *Leishmania donovani*. PLoS Negl Trop Dis 14:e0007143. doi:10.1371/journal.pntd.0007143. 32310945 PMC7237039

[24] Gelanew T, Hailu A, Schőnian G, Lewis MD, Miles MA, Yeo M. 2014. Multilocus sequence and microsatellite identification of intra-specific hybrids and ancestor-like donors among natural Ethiopian isolates of *Leishmania donovani*. Int J Parasitol 44:751–757. doi:10.1016/j.ijpara.2014.05.008. 24995620 PMC4147965

[25] Takele Y, Mulaw T, Adem E, Shaw CJ, Franssen SU, Womersley R, Kaforou M, Taylor GP, Levin M, Müller I, Cotton JA, Kropf P. 1 4 2021. Immunological factors, but not clinical features, predict visceral leishmaniasis relapse in patients co-infected with HIV. bioRxiv doi:10.1101/2021.03.30.437646. PMC8784791 35106507

[26] Sterkers Y, Lachaud L, Bourgeois N, Crobu L, Bastien P, Pages M. 2012. Novel insights into genome plasticity in Eukaryotes: mosaic aneuploidy in *Leishmania*. Mol Microbiol 86:15–23. doi:10.1111/j.1365-2958.2012.08185.x. 22857263

[27] Dumetz F, Imamura H, Sanders M, Seblova V, Myskova J, Pescher P, Vanaerschot M, Meehan CJ, Cuypers B, De Muylder G, Spath GF, Bussotti G, Vermeesch JR, Berriman M, Cotton JA, Volf P, Dujardin JC, Domagalska MA. 2017. Modulation of aneuploidy in *Leishmania donovani* during adaptation to different *in vitro* and *in vivo* environments and its impact on gene expression. mBio 8:e00599-17. doi:10.1128/mBio.00599-17. 28536289 PMC5442457

[28] Callahan HL, Beverley SM. 1991. Heavy metal resistance: a new role for P-glycoproteins in *Leishmania*. J Biol Chem 266:18427–18430. doi:10.1016/S0021-9258(18)55077-8. 1680861

[29] Ashutosh Garg M, Sundar S, Duncan R, Nakhasi HL, Goyal N. 2012. Downregulation of mitogen-activated protein kinase 1 of *Leishmania donovani* field isolates is associated with antimony resistance. Antimicrob Agents Chemother 56:518–525. doi:10.1128/AAC.00736-11. 22064540 PMC3256019

[30] Perez-Victoria FJ, Gamarro F, Ouellette M, Castanys S. 2003. Functional cloning of the miltefosine transporter. A novel P-type phospholipid translocase from *Leishmania* involved in drug resistance. J Biol Chem 278:49965–49971. doi:10.1074/jbc.M308352200. 14514670

[31] Carnielli JBT, Crouch K, Forrester S, Silva VC, Carvalho SFG, Damasceno JD, Brown E, Dickens NJ, Costa DL, Costa CHN, Dietze R, Jeffares DC, Mottram JC. 2018. A *Leishmania infantum* genetic marker associated with miltefosine treatment failure for visceral leishmaniasis. EBioMedicine 36:83–91. doi:10.1016/j.ebiom.2018.09.029. 30268832 PMC6197651

[32] Prieto Barja P, Pescher P, Bussotti G, Dumetz F, Imamura H, Kedra D, Domagalska M, Chaumeau V, Himmelbauer H, Pages M, Sterkers Y, Dujardin JC, Notredame C, Spath GF. 2017. Haplotype selection as an adaptive mechanism in the protozoan pathogen *Leishmania donovani*. Nat Ecol Evol 1:1961–1969. doi:10.1038/s41559-017-0361-x. 29109466

[33] Tiruneh K, Wasie B, Gonzalez H. 2015. Sexual behavior and vulnerability to HIV infection among seasonal migrant laborers in Metema district, northwest Ethiopia: a cross-sectional study. BMC Public Health 15:122. doi:10.1186/s12889-015-1468-0. 25885580 PMC4330642

[34] Mandell MA, Beverley SM. 2017. Continual renewal and replication of persistent *Leishmania* major parasites in concomitantly immune hosts. Proc Natl Acad Sci U S A 114:E801–E810. doi:10.1073/pnas.1619265114. 28096392 PMC5293024

[35] Mendonca MG, de Brito ME, Rodrigues EH, Bandeira V, Jardim ML, Abath FG. 2004. Persistence of leishmania parasites in scars after clinical cure of American cutaneous leishmaniasis: is there a sterile cure? J Infect Dis 189:1018–1023. doi:10.1086/382135. 14999605

[36] Hossain F, Ghosh P, Khan MAA, Duthie MS, Vallur AC, Picone A, Howard RF, Reed SG, Mondal D. 2017. Real-time PCR in detection and quantitation of *Leishmania donovani* for the diagnosis of visceral leishmaniasis patients and the monitoring of their response to treatment. PLoS One 12:e0185606. doi:10.1371/journal.pone.0185606. 28957391 PMC5619796

[37] Czechowicz RT, Millard TP, Smith HR, Ashton RE, Lucas SB, Hay RJ. 1999. Reactivation of cutaneous leishmaniasis after surgery. Br J Dermatol 141:1113–1116. doi:10.1046/j.1365-2133.1999.03215.x. 10606863

[38] Simon I, Wissing KM, Del Marmol V, Antinori S, Remmelink M, Nilufer Broeders E, Nortier JL, Corbellino M, Abramowicz D, Cascio A. 2011. Recurrent leishmaniasis in kidney transplant recipients: report of 2 cases and systematic review of the literature. Transpl Infect Dis 13:397–406. doi:10.1111/j.1399-3062.2011.00598.x. 21281418

[39] Broeckaert-van Orshoven A, Michielsen P, Vandepitte J. 1979. Fatal leishmaniasis in renal-transplant patient. Lancet 2:740–741. doi:10.1016/s0140-6736(79)90664-0. 90824

[40] Sacks DL. 2014. Vaccines against tropical parasitic diseases: a persisting answer to a persisting problem. Nat Immunol 15:403–405. doi:10.1038/ni.2853. 24747701 PMC4814932

[41] Kirstein OD, Abbasi I, Horwitz BZ, Skrip L, Hailu A, Jaffe C, Li LL, Prow TW, Warburg A. 2017. Minimally invasive microbiopsies: a novel sampling method for identifying asymptomatic, potentially infectious carriers of Leishmania donovani. Int J Parasitol 47:609–616. doi:10.1016/j.ijpara.2017.02.005. 28455239 PMC5596977

[42] Zackay A, Cotton JA, Sanders M, Hailu A, Nasereddin A, Warburg A, Jaffe CL. 2018. Genome wide comparison of Ethiopian *Leishmania donovani* strains reveals differences potentially related to parasite survival. PLoS Genet 14:e1007133. doi:10.1371/journal.pgen.1007133. 29315303 PMC5777657

[43] Cupolillo E, Cavalcanti AS, Ferreira GEM, Boite MC, Morgado FN, Porrozzi R. 2020. Occurrence of multiple genotype infection caused by *Leishmania infantum* in naturally infected dogs. PLoS Negl Trop Dis 14:e0007986. doi:10.1371/journal.pntd.0007986. 32716941 PMC7410330

[44] Domagalska MA, Imamura H, Sanders M, Van den Broeck F, Bhattarai NR, Vanaerschot M, Maes I, D'Haenens E, Rai K, Rijal S, Berriman M, Cotton JA, Dujardin JC. 2019. Genomes of *Leishmania* parasites directly sequenced from patients with visceral leishmaniasis in the Indian subcontinent. PLoS Negl Trop Dis 13:e0007900. doi:10.1371/journal.pntd.0007900. 31830038 PMC6932831

[45] Parvizi P, Baghban N, Novin EA, Absavaran A. 2010. Detection, identification and molecular typing of *Leishmania* major in *Phlebotomus papatasi* from a focus of zoonotic cutaneous leishmaniasis in central of Iran. Exp Parasitol 124:232–237. doi:10.1016/j.exppara.2009.10.004. 19854172

[46] Darvishi M, Yaghoobi-Ershadi MR, Shahbazi F, Akhavan AA, Jafari R, Soleimani H, Yaghoobi-Ershadi N, Khajeian M, Darabi H, Arandian MH. 2015. Epidemiological study on sand flies in an endemic focus of cutaneous leishmaniasis, Bushehr city, southwestern Iran. Front Public Health 3:14. doi:10.3389/fpubh.2015.00014. 25699245 PMC4313593

[47] Serafim TD, Coutinho-Abreu IV, Oliveira F, Meneses C, Kamhawi S, Valenzuela JG. 2018. Sequential blood meals promote *Leishmania* replication and reverse metacyclogenesis augmenting vector infectivity. Nat Microbiol 3:548–555. doi:10.1038/s41564-018-0125-7. 29556108 PMC6007031

[48] Cameron MM, Acosta-Serrano A, Bern C, Boelaert M, den Boer M, Burza S, Chapman LA, Chaskopoulou A, Coleman M, Courtenay O, Croft S, Das P, Dilger E, Foster G, Garlapati R, Haines L, Harris A, Hemingway J, Hollingsworth TD, Jervis S, Medley G, Miles M, Paine M, Picado A, Poche R, Ready P, Rogers M, Rowland M, Sundar S, de Vlas SJ, Weetman D. 2016. Understanding the transmission dynamics of *Leishmania donovani* to provide robust evidence for interventions to eliminate visceral leishmaniasis in Bihar, India. Parasit Vectors 9:25. doi:10.1186/s13071-016-1309-8. 26812963 PMC4729074

[49] Fuller GK, Lemma A, Haile T, Atwood CL. 1976. Kala-azar in Ethopia I: leishmanin skin test in Setit Humera, a kala-azar endemic area in northwestern Ethiopia. Ann Trop Med Parasitol 70:147–163. doi:10.1080/00034983.1976.11687108. 938122

[50] van Griensven J, van Henten S, Mengesha B, Kassa M, Adem E, Endris Seid M, Abdellati S, Asefa W, Simegn T, Debasu D, Bogale T, Gedamu Y, Van Den Bossche D, Adriaensen W, Van der Auwera G, Cnops L, Vogt F, Diro E. 2019. Longitudinal evaluation of asymptomatic *Leishmania* infection in HIV-infected individuals in north-west Ethiopia: a pilot study. PLoS Negl Trop Dis 13:e0007765. doi:10.1371/journal.pntd.0007765. 31593563 PMC6799935

[51] Ferede G, Diro E, Getie S, Getnet G, Takele Y, Amsalu A, Wondimeneh Y. 2017. Visceral leishmaniasis-malaria coinfection and their associated factors in patients attending Metema Hospital, northwest Ethiopia: suggestion for integrated vector management. Malar Res Treat 2017:6816913. doi:10.1155/2017/6816913. 28932617 PMC5592390

[52] Kirstein OD, Skrip L, Abassi I, Iungman T, Horwitz BZ, Gebresilassie A, Spitzova T, Waitz Y, Gebre-Michael T, Volf P, Hailu A, Warburg A. 2018. A fine scale eco-epidemiological study on endemic visceral leishmaniasis in north Ethiopian villages. Acta Trop 183:64–77. doi:10.1016/j.actatropica.2018.04.005. 29621537 PMC5956276

[53] Guggisberg AM, Sundararaman SA, Lanaspa M, Moraleda C, Gonzalez R, Mayor A, Cistero P, Hutchinson D, Kremsner PG, Hahn BH, Bassat Q, Odom AR. 2016. Whole-genome sequencing to evaluate the resistance landscape following antimalarial treatment failure with fosmidomycin-clindamycin. J Infect Dis 214:1085–1091. doi:10.1093/infdis/jiw304. 27443612 PMC5021231

[54] Popovici J, Friedrich LR, Kim S, Bin S, Run V, Lek D, Cannon MV, Menard D, Serre D. 2018. Genomic analyses reveal the common occurrence and complexity of *Plasmodium vivax* relapses in Cambodia. mBio 9:e01888-17. doi:10.1128/mBio.01888-17. 29362233 PMC5784252

[55] Rutledge GG, Marr I, Huang GKL, Auburn S, Marfurt J, Sanders M, White NJ, Berriman M, Newbold CI, Anstey NM, Otto TD, Price RN. 2017. Genomic characterization of recrudescent *Plasmodium malariae* after treatment with artemether/lumefantrine. Emerg Infect Dis 23:1300–1307. doi:10.3201/eid2308.161582. 28430103 PMC5547787

[56] Hide M, Singh R, Kumar B, Banuls AL, Sundar S. 2007. A microculture technique for isolating live *Leishmania* parasites from peripheral blood of visceral leishmaniasis patients. Acta Trop 102:197–200. doi:10.1016/j.actatropica.2007.04.015. 17544353

[57] Dereure J, Pratlong F, Reynes J, Basset D, Bastien P, Dedet JP. 1998. Haemoculture as a tool for diagnosing visceral leishmaniasis in HIV-negative and HIV-positive patients: interest for parasite identification. Bull World Health Organ 76:203–206. 9648362 PMC2305644

[58] Sudarshan M, Singh T, Chakravarty J, Sundar S. 2015. A correlative study of splenic parasite score and peripheral blood parasite load estimation by quantitative PCR in visceral leishmaniasis. J Clin Microbiol 53:3905–3907. doi:10.1128/JCM.01465-15. 26400788 PMC4652099

[59] Akopyants NS, Kimblin N, Secundino N, Patrick R, Peters N, Lawyer P, Dobson DE, Beverley SM, Sacks DL. 2009. Demonstration of genetic exchange during cyclical development of *Leishmania* in the sand fly vector. Science 324:265–268. doi:10.1126/science.1169464. 19359589 PMC2729066

[60] Rogers MB, Downing T, Smith BA, Imamura H, Sanders M, Svobodova M, Volf P, Berriman M, Cotton JA, Smith DF. 2014. Genomic confirmation of hybridisation and recent inbreeding in a vector-isolated *Leishmania* population. PLoS Genet 10:e1004092. doi:10.1371/journal.pgen.1004092. 24453988 PMC3894156

[61] Sterkers Y, Crobu L, Lachaud L, Pages M, Bastien P. 2014. Parasexuality and mosaic aneuploidy in *Leishmania*: alternative genetics. Trends Parasitol 30:429–435. doi:10.1016/j.pt.2014.07.002. 25073852

[62] Mannaert A, Downing T, Imamura H, Dujardin JC. 2012. Adaptive mechanisms in pathogens: universal aneuploidy in *Leishmania*. Trends Parasitol 28:370–376. doi:10.1016/j.pt.2012.06.003. 22789456

[63] Downing T, Imamura H, Decuypere S, Clark TG, Coombs GH, Cotton JA, Hilley JD, de Doncker S, Maes I, Mottram JC, Quail MA, Rijal S, Sanders M, Schonian G, Stark O, Sundar S, Vanaerschot M, Hertz-Fowler C, Dujardin JC, Berriman M. 2011. Whole genome sequencing of multiple *Leishmania donovani* clinical isolates provides insights into population structure and mechanisms of drug resistance. Genome Res 21:2143–2156. doi:10.1101/gr.123430.111. 22038251 PMC3227103

[64] Imamura H, Downing T, Van den Broeck F, Sanders MJ, Rijal S, Sundar S, Mannaert A, Vanaerschot M, Berg M, De Muylder G, Dumetz F, Cuypers B, Maes I, Domagalska M, Decuypere S, Rai K, Uranw S, Bhattarai NR, Khanal B, Prajapati VK, Sharma S, Stark O, Schonian G, De Koning HP, Settimo L, Vanhollebeke B, Roy S, Ostyn B, Boelaert M, Maes L, Berriman M, Dujardin JC, Cotton JA. 2016. Evolutionary genomics of epidemic visceral leishmaniasis in the Indian subcontinent. Elife 5:e12613. doi:10.7554/eLife.12613. 27003289 PMC4811772

[65] Patino LH, Muskus C, Munoz M, Ramirez JD. 2020. Genomic analyses reveal moderate levels of ploidy, high heterozygosity and structural variations in a Colombian isolate of *Leishmania* (*Leishmania*) *amazonensis*. Acta Trop 203:105296. doi:10.1016/j.actatropica.2019.105296. 31836281

[66] Imamura H, Monsieurs P, Jara M, Sanders M, Maes I, Vanaerschot M, Berriman M, Cotton JA, Dujardin JC, Domagalska MA. 2020. Evaluation of whole genome amplification and bioinformatic methods for the characterization of Leishmania genomes at a single cell level. Sci Rep 10:15043. doi:10.1038/s41598-020-71882-2. 32929126 PMC7490275

[67] Yardley V, Ortuno N, Llanos-Cuentas A, Chappuis F, Doncker SD, Ramirez L, Croft S, Arevalo J, Adaui V, Bermudez H, Decuypere S, Dujardin JC. 2006. American tegumentary leishmaniasis: is antimonial treatment outcome related to parasite drug susceptibility? J Infect Dis 194:1168–1175. doi:10.1086/507710. 16991093

[68] Ponte-Sucre A, Gamarro F, Dujardin JC, Barrett MP, Lopez-Velez R, Garcia-Hernandez R, Pountain AW, Mwenechanya R, Papadopoulou B. 2017. Drug resistance and treatment failure in leishmaniasis: a 21st century challenge. PLoS Negl Trop Dis 11:e0006052. doi:10.1371/journal.pntd.0006052. 29240765 PMC5730103

[69] Rijal S, Yardley V, Chappuis F, Decuypere S, Khanal B, Singh R, Boelaert M, De Doncker S, Croft S, Dujardin JC. 2007. Antimonial treatment of visceral leishmaniasis: are current *in vitro* susceptibility assays adequate for prognosis of *in vivo* therapy outcome? Microbes Infect 9:529–535. doi:10.1016/j.micinf.2007.01.009. 17350306

[70] Croft SL, Sundar S, Fairlamb AH. 2006. Drug resistance in leishmaniasis. Clin Microbiol Rev 19:111–126. doi:10.1128/CMR.19.1.111-126.2006. 16418526 PMC1360270

[71] Sunyoto T, Potet J, Boelaert M. 2018. Why miltefosine-a life-saving drug for leishmaniasis-is unavailable to people who need it the most. BMJ Glob Health 3:e000709. doi:10.1136/bmjgh-2018-000709. PMC5935166 29736277

[72] Gidey K, Belay D, Hailu BY, Kassa TD, Niriayo YL. 2019. Visceral leishmaniasis treatment outcome and associated factors in northern Ethiopia. Biomed Res Int 2019:3513957. doi:10.1155/2019/3513957. 31531350 PMC6719273

[73] Monge-Maillo B, Lopez-Velez R. 2016. Treatment options for visceral leishmaniasis and HIV coinfection. AIDS Rev 18:32–43. 26936761

[74] Pescher P, Blisnick T, Bastin P, Spath GF. 2011. Quantitative proteome profiling informs on phenotypic traits that adapt *Leishmania donovani* for axenic and intracellular proliferation. Cell Microbiol 13:978–991. doi:10.1111/j.1462-5822.2011.01593.x. 21501362

[75] Kozarewa I, Ning Z, Quail MA, Sanders MJ, Berriman M, Turner DJ. 2009. Amplification-free Illumina sequencing-library preparation facilitates improved mapping and assembly of (G+C)-biased genomes. Nat Methods 6:291–295. doi:10.1038/nmeth.1311. 19287394 PMC2664327

[76] Bolger AM, Lohse M, Usadel B. 2014. Trimmomatic: a flexible trimmer for Illumina sequence data. Bioinformatics 30:2114–2120. doi:10.1093/bioinformatics/btu170. 24695404 PMC4103590

[77] Li H, Durbin R. 2009. Fast and accurate short read alignment with Burrows-Wheeler transform. Bioinformatics 25:1754–1760. doi:10.1093/bioinformatics/btp324. 19451168 PMC2705234

[78] Van der Auwera GA, Carneiro MO, Hartl C, Poplin R, Del AG, Levy-Moonshine A, Jordan T, Shakir K, Roazen D, Thibault J, Banks E, Garimella KV, Altshuler D, Gabriel S, DePristo MA. 2013. From FastQ data to high confidence variant calls: the Genome Analysis Toolkit best practices pipeline. Curr Protoc Bioinformatics 43:11.10.1–11.10.33. doi:10.1002/0471250953.bi1110s43. 25431634 PMC4243306

[79] Poplin R, Ruano-Rubio V, DePristo MA, Fennell TJ, Carneiro MO, Van der Auwera G, Kling DE, Gauthier LD, Levy-Moonshine A, Roazen D, Shakir K, Thibault J, Chandran S, Whelan C, Lek M, Gabriel S, Daly MJ, Neale B, MacArthur DG, Banks E. 24 7 2018. Scaling accurate genetic variant discovery to tens of thousands of samples. bioRxiv doi:10.1101/201178.

[80] Pembleton LW, Cogan NO, Forster JW. 2013. StAMPP: an R package for calculation of genetic differentiation and structure of mixed-ploidy level populations. Mol Ecol Resour 13:946–952. doi:10.1111/1755-0998.12129. 23738873

[81] Paradis E, Schliep K. 2019. ape 5.0: an environment for modern phylogenetics and evolutionary analyses in R. Bioinformatics 35:526–528. doi:10.1093/bioinformatics/bty633. 30016406

[82] Blomberg SP, Garland T, Jr, Ives AR. 2003. Testing for phylogenetic signal in comparative data: behavioral traits are more labile. Evolution 57:717–745. doi:10.1111/j.0014-3820.2003.tb00285.x. 12778543

[83] R Core Development Team. 2017. R: a language and environment for statistical computing. R Foundation for Statistical Computing, Vienna, Austria.

[84] Shaw CD, Lonchamp J, Downing T, Imamura H, Freeman TM, Cotton JA, Sanders M, Blackburn G, Dujardin JC, Rijal S, Khanal B, Illingworth CJ, Coombs GH, Carter KC. 2016. *In vitro* selection of miltefosine resistance in promastigotes of *Leishmania donovani* from Nepal: genomic and metabolomic characterization. Mol Microbiol 99:1134–1148. doi:10.1111/mmi.13291. 26713880 PMC4832254

[85] Perez-Victoria FJ, Sanchez-Canete MP, Castanys S, Gamarro F. 2006. Phospholipid translocation and miltefosine potency require both *L. donovani* miltefosine transporter and the new protein LdRos3 in *Leishmania* parasites. J Biol Chem 281:23766–23775. doi:10.1074/jbc.M605214200. 16785229

