# Supplementary material for: Diversity and Within-Host Evolution of Leishmania donovani from Visceral Leishmaniasis Patients with and without HIV Coinfection in Northern Ethiopia
Source: mBio. 2021 Jun 29;12(3):e00971-21. doi: 10.1128/mBio.00971-21 (PMC8262925; doi:10.1128/mBio.00971-21)
Supplement: FIG S1 [file mbio.00971-21-sf001.pdf]

Fig. S1 Genomic relatedness between first parasite isolates taken from each patient of primary VL only.

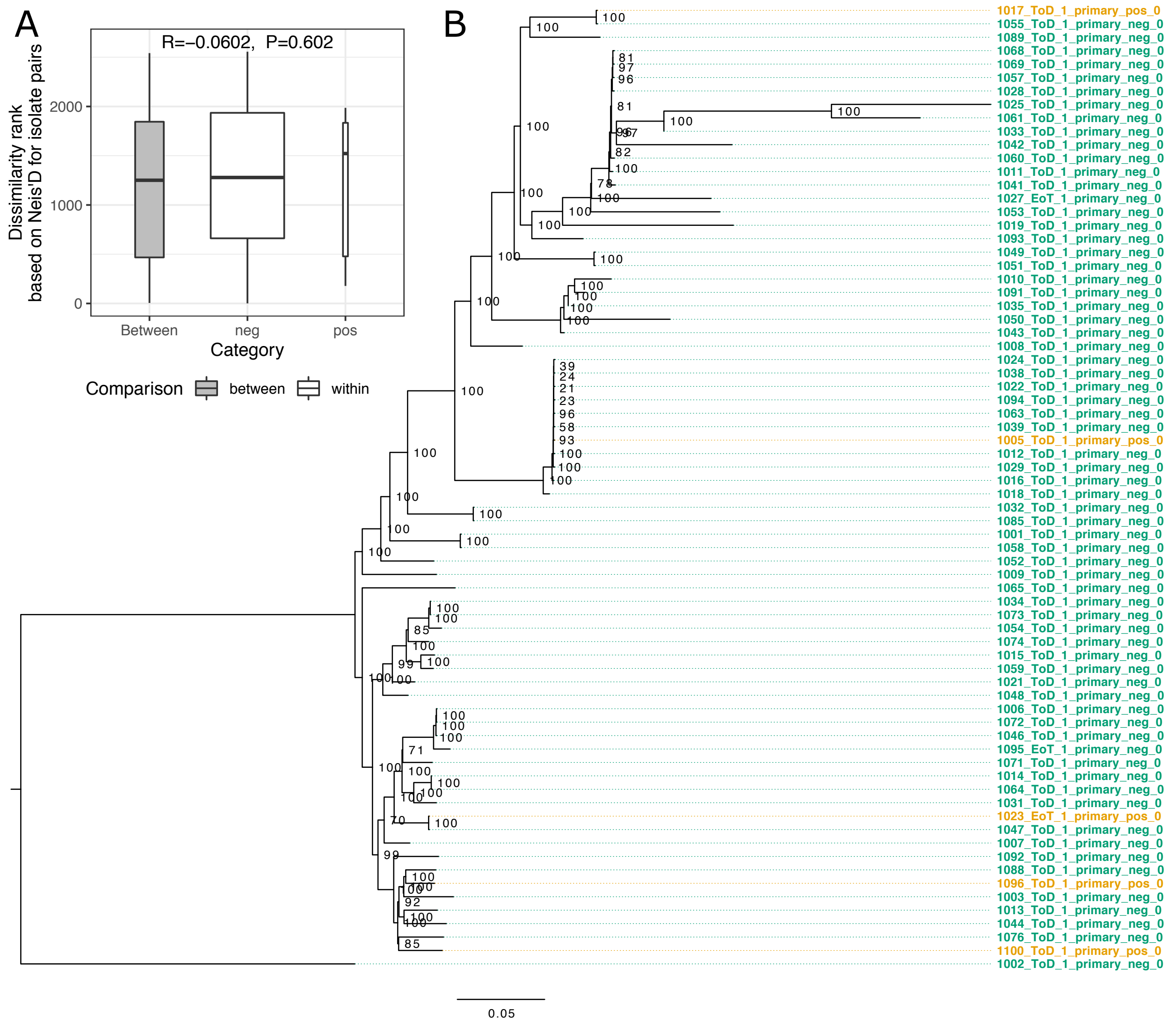

Figure S1. Genomic relatedness between first parasite isolates taken from each patient of primary VL only. A) ANOSIM results comparing sum of ranked pairwise genetic distances (Nei's D) within and between HIV positive and negative samples from primary VL only. B) Phylogeny of first isolate of primary infection taken from each patient. Sample colour indicates HIV status: green for isolates from HIV negative patients, light orange for HIV positive. Sample names are composed as in figure 1. Bootstrap values are indicated at branch nodes. The phylogeny is rooted based on the inclusion of an *L. infantum* outgroup (data not shown).
