# Supplementary material for: Diversity and Within-Host Evolution of Leishmania donovani from Visceral Leishmaniasis Patients with and without HIV Coinfection in Northern Ethiopia
Source: mBio. 2021 Jun 29;12(3):e00971-21. doi: 10.1128/mBio.00971-21 (PMC8262925; doi:10.1128/mBio.00971-21)
Supplement: FIG S2 [file mbio.00971-21-sf002.pdf]

Fig. S2 Heatmap of Nei's distances for isolates of patients 1023 and 1045.

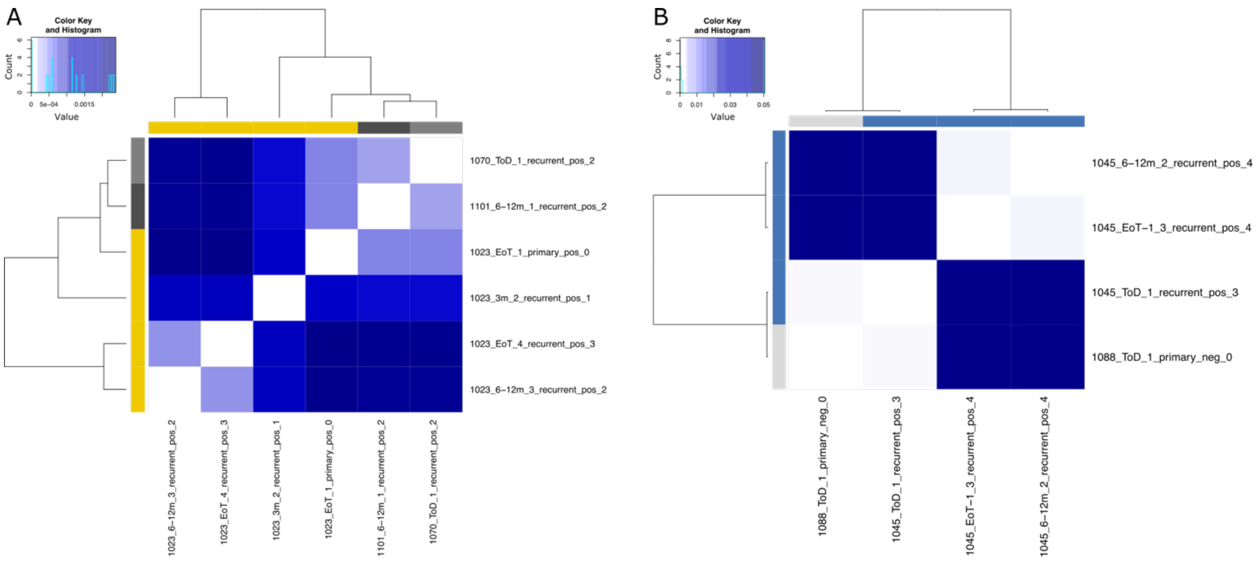

Figure S2. Heatmap of Nei's distances for isolates of patients 1023 and 1045. Heatmaps display pairwise distances between all samples of the respective patient from different time points of isolation and the closest sample from our entire sample collection if closer than the remaining isolates from the same patient. A) Four samples from patient 1023 isolated from primary VL and recurrent VL relapses 1, 2 and 3 are shown along with two isolates from different patients closest to the isolate from primary VL. Time series isolates diverge gradually with time. B) Three samples from patient 1045 were isolated at recurrent VL "relapse 3" and "relapse 4" before and after treatment. The isolate from recurrent VL "relapse 3" was closest to an isolate from another patient and is very different from the two isolates from the subsequent recurrent VL relapse.
