# Supplementary material for: Diversity and Within-Host Evolution of Leishmania donovani from Visceral Leishmaniasis Patients with and without HIV Coinfection in Northern Ethiopia
Source: mBio. 2021 Jun 29;12(3):e00971-21. doi: 10.1128/mBio.00971-21 (PMC8262925; doi:10.1128/mBio.00971-21)
Supplement: FIG S3 [file mbio.00971-21-sf003.pdf]

Fig. S3 Relationship of the heterozygous fraction with other disease phenotypes.

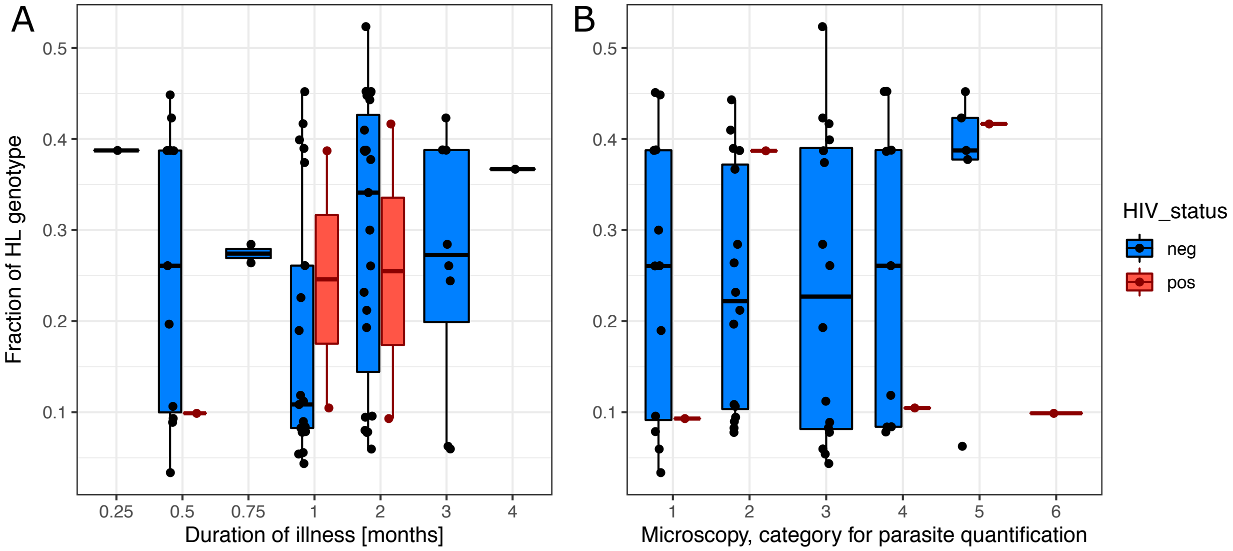

Figure S3. Relationship of the heterozygous fraction with other disease phenotypes. The fraction of heterozygotes in an isolate with respect to the A) duration of symptomatic primary VL and B) parasite load at the time of hospital acception for symptomatic primary VL. Linear models for both phenotypes of interest did not show any linear relationship with duration of illness or parasite load, respectively. Neither was HIV status an explanatory factor in either case. A) lm(fHL ~ duration_illness_months + HIV_status, duration_illness_months: estimate=0.0305, p-value=0.151, HIV_status: estimate=-0.0231, p-value=0.741) B) lm(fHL ~ microscopy + HIV_status, microscopy: estimate=0.0127, p-value=0.403, HIV_status: estimate=-0.0420, p-value=0.565).
