# Supplementary material for: Diversity and Within-Host Evolution of Leishmania donovani from Visceral Leishmaniasis Patients with and without HIV Coinfection in Northern Ethiopia
Source: mBio. 2021 Jun 29;12(3):e00971-21. doi: 10.1128/mBio.00971-21 (PMC8262925; doi:10.1128/mBio.00971-21)
Supplement: FIG S4 [file mbio.00971-21-sf004.pdf]

Fig. S4 Use of allele frequency estimates to evaluate clonal diversity of individual isolates.

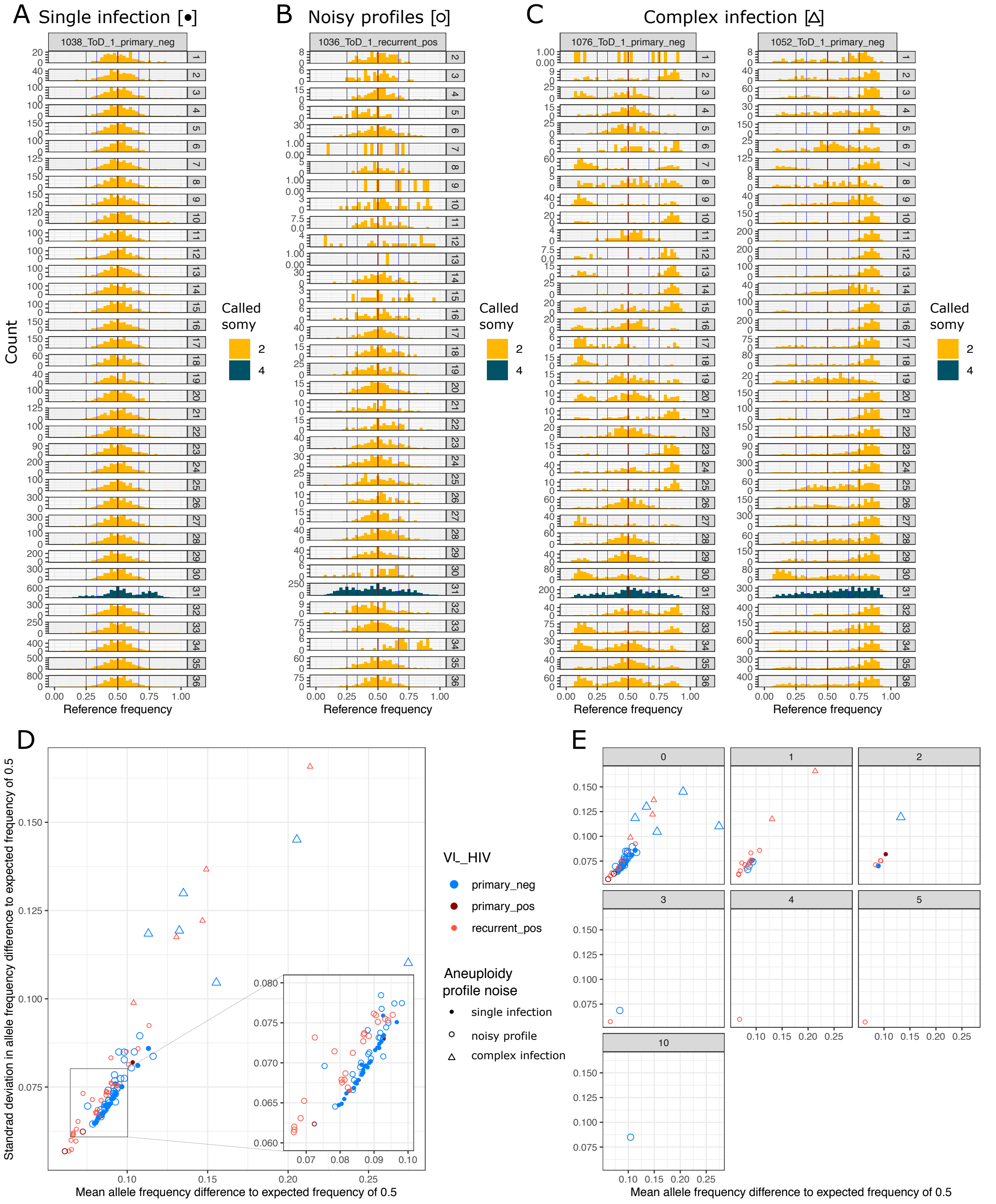

Figure S4. Use of allele frequency estimates to evaluate clonal diversity of individual isolates. Plots A-C display one or two examples of categories for the noisiness of aneuploidy profiles based

on allele frequency estimates for each isolate. Categories were determined by visual inspection of the allele frequency plots by chromosome for each isolate. A) Single infection: allele frequency distributions across chromosomes peak at the frequency expected based on the chromosome somy without outliers from the distribution around the peak. B) Noisy profiles: while allele frequency distributions mainly peak around the expected mean, some noise is visible. C) Complex infection: allele frequency distributions have multiple peaks, also at high and low frequency. D) Mean and standard deviation (sd) across isolates from expected allele frequency of 0.5 for diploid chromosomes. Symbols are coloured by VL/HIV status. Symbol types display the category of the noise of the aneuploidy profile as shown in A-C. The visual categorisation is partly mirrored by the mean and sd of the deviation from the allele frequency expectation. E) Data from A is shown in seven different subplots that indicate the summed difference of the isolate's aneuploidy profile from the common aneuploidy profile (diploid for all chromosomes except for a tetraploid chromosome 31). Summed up differences are indicated at the top of each subplot.
