# Supplementary material for: Diversity and Within-Host Evolution of Leishmania donovani from Visceral Leishmaniasis Patients with and without HIV Coinfection in Northern Ethiopia
Source: mBio. 2021 Jun 29;12(3):e00971-21. doi: 10.1128/mBio.00971-21 (PMC8262925; doi:10.1128/mBio.00971-21)
Supplement: FIG S5 [file mbio.00971-21-sf005.pdf]

Fig. S5 Allele frequency profiles for isolates from patient 1045.

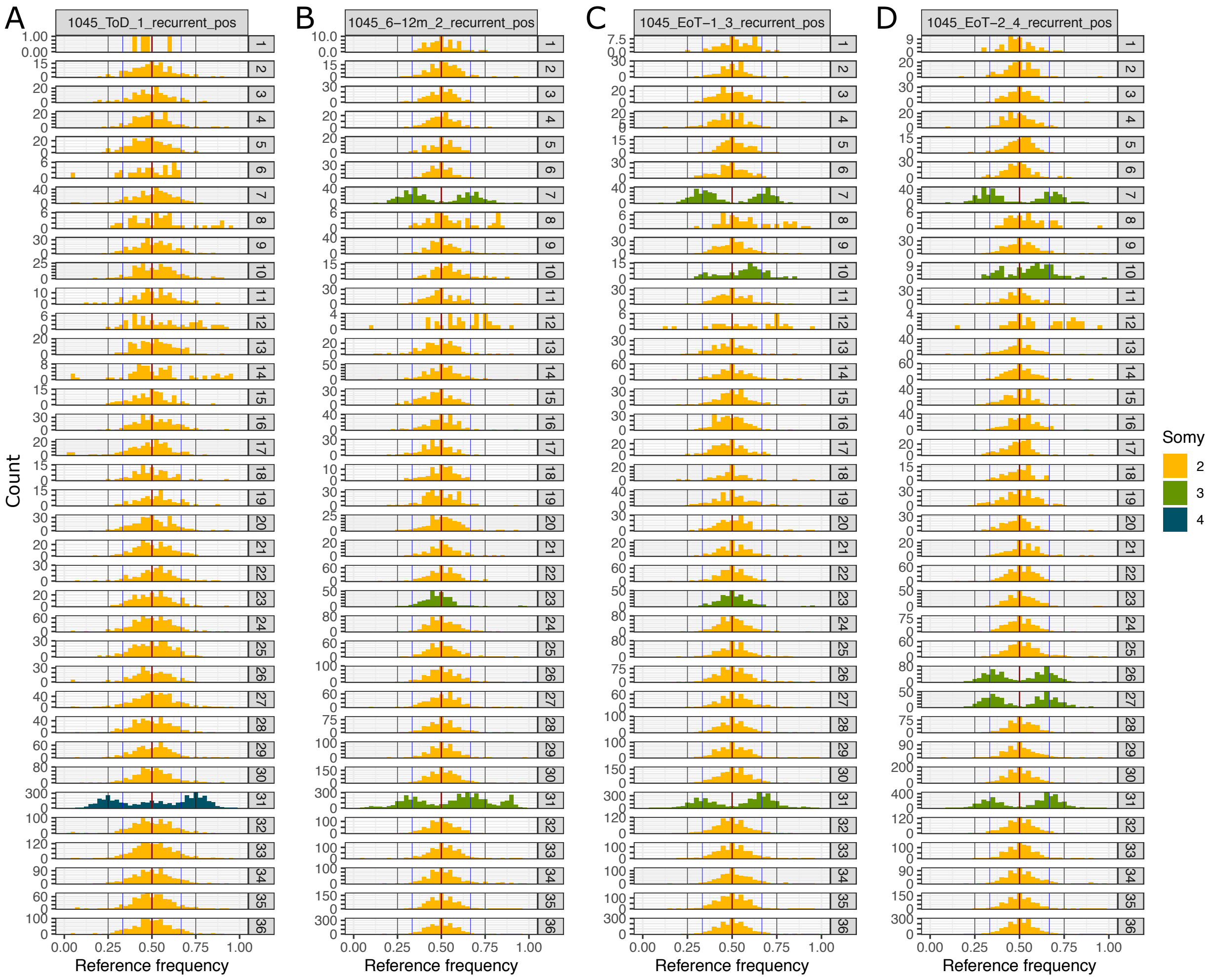

Figure S5. Allele frequency profiles for isolates from patient 1045. Allele frequency profiles are shown separately for each chromosome and isolate and color coded by the respective chromosome copy number. All profiles have been categorised as noisy though they dominantly follow an expected frequency distribution based on respective somies (Fig. S4).
