# Supplementary material for: Diversity and Within-Host Evolution of Leishmania donovani from Visceral Leishmaniasis Patients with and without HIV Coinfection in Northern Ethiopia
Source: mBio. 2021 Jun 29;12(3):e00971-21. doi: 10.1128/mBio.00971-21 (PMC8262925; doi:10.1128/mBio.00971-21)
Supplement: FIG S6 [file mbio.00971-21-sf006.pdf]

Fig. S6 Aneuploidy profiles of all 113 parasite samples.

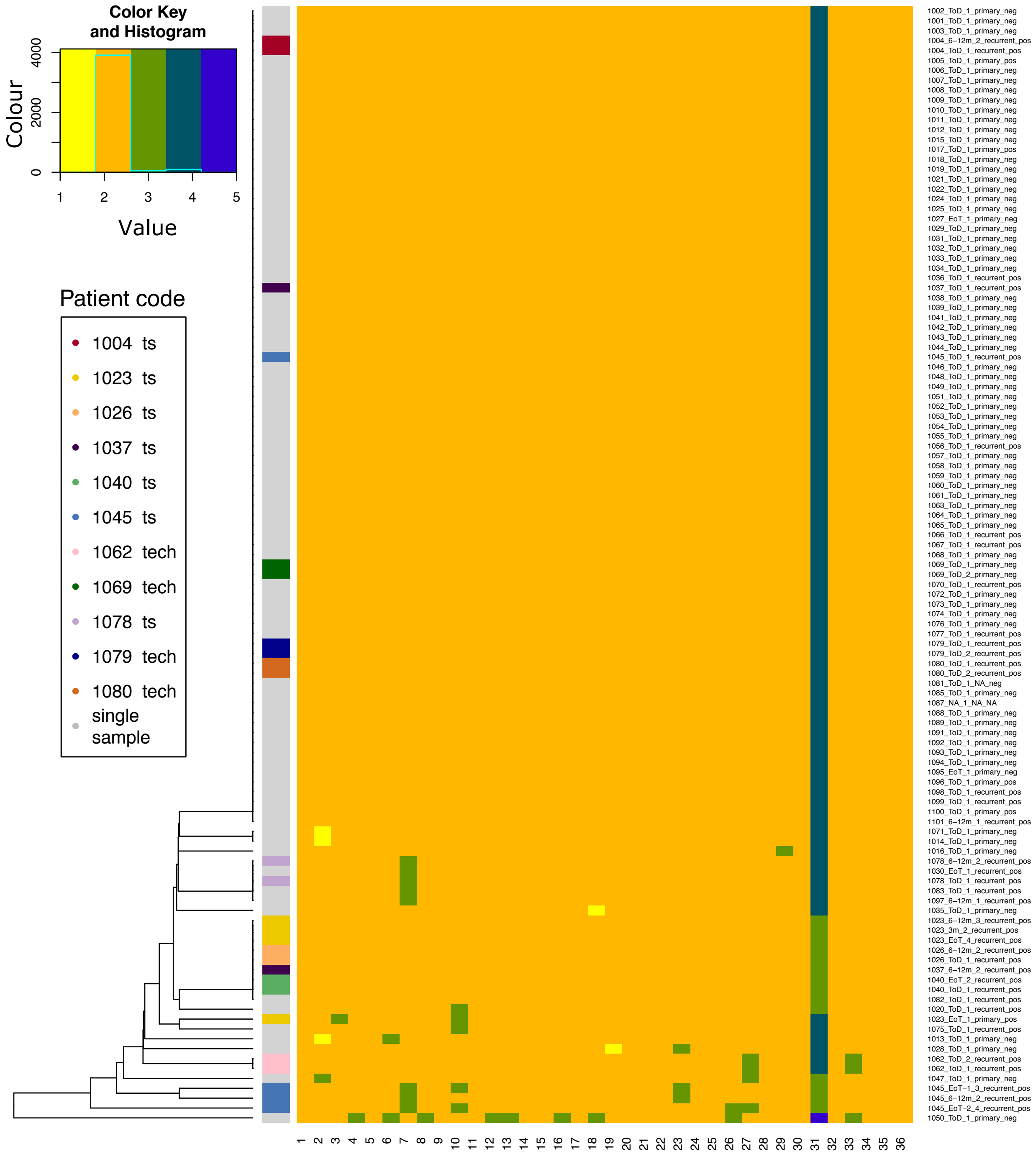

Figure S6. Aneuploidy profiles of all 113 parasite samples. The heatmap displays aneuploidy profiles with color coded somies for each patient (rows) and chromosome (columns). Sample identifiers are listed on the right-hand side of the respective row. The leftmost colour column indicates patient association of patients with time series data (ts) and /or isolate aliquots (tech) using different colors for each patient. Samples from all remaining patients with a single isolate

only are coloured in gray. Rows with aneuploidy profiles are ordered with average linkage clustering with the cladogram shown on the left.
