# Supplementary material for: Diversity and Within-Host Evolution of Leishmania donovani from Visceral Leishmaniasis Patients with and without HIV Coinfection in Northern Ethiopia
Source: mBio. 2021 Jun 29;12(3):e00971-21. doi: 10.1128/mBio.00971-21 (PMC8262925; doi:10.1128/mBio.00971-21)
Supplement: TABLE S2 [file mbio.00971-21-st002.pdf]

Table S2. Aneuploidy profiles and metadata summary of all isolates from patients with time series data and /or replicates of primary isolates.

Profile identifier	Profile count	Patient code	Sample [patient code, study timepoint, sample no for patient, VL category, HIV status]	Aneuploidy profile [differences to most common profile: all chrs are 2, except chr 31 with 4]	Replicate of primary isolate
1	83	1004	1004_ToD_1_recurrent_pos	------------------------------------	FALSE
1	83	1004	1004_6-12m_2_recurrent_pos	------------------------------------	FALSE
16	1	1023	1023_EoT_1_primary_pos	--1------1--------------------------	FALSE
2	9	1023	1023_3m_2_recurrent_pos	------------------------------1-----	FALSE
2	9	1023	1023_6-12m_3_recurrent_pos	------------------------------1-----	FALSE
2	9	1023	1023_EoT_4_recurrent_pos	------------------------------1-----	FALSE
2	9	1026	1026_ToD_1_recurrent_pos	------------------------------1-----	FALSE
2	9	1026	1026_6-12m_2_recurrent_pos	------------------------------1-----	FALSE
1	83	1037	1037_ToD_1_recurrent_pos	------------------------------------	FALSE
2	9	1037	1037_6-12m_2_recurrent_pos	------------------------------1-----	FALSE
2	9	1040	1040_ToD_1_recurrent_pos	------------------------------1-----	FALSE
2	9	1040	1040_EoT_2_recurrent_pos	------------------------------1-----	FALSE
1	83	1045	1045_ToD_1_recurrent_pos	------------------------------------	FALSE
12	1	1045	1045_6-12m_2_recurrent_pos*	------1---------------1-------1-----	FALSE
14	1	1045	1045_EoT-1_3_recurrent_pos*	------1--1------------1-------1-----	FALSE
13	1	1045	1045_EoT-2_4_recurrent_pos*	------1--1---------------11---1-----	TRUE
5	2	1062	1062_ToD_1_recurrent_pos	--------------------------1-----1---	FALSE
5	2	1062	1062_ToD_2_recurrent_pos	--------------------------1-----1---	TRUE
1	83	1069	1069_ToD_1_primary_neg	------------------------------------	FALSE
1	83	1069	1069_ToD_2_primary_neg	------------------------------------	TRUE
3	5	1078	1078_ToD_1_recurrent_pos	------1-----------------------------	FALSE
3	5	1078	1078_6-12m_2_recurrent_pos	------1-----------------------------	FALSE
1	83	1079	1079_ToD_1_recurrent_pos	------------------------------------	FALSE
1	83	1079	1079_ToD_2_recurrent_pos	------------------------------------	TRUE
1	83	1080	1080_ToD_1_recurrent_pos	------------------------------------	FALSE
1	83	1080	1080_ToD_2_recurrent_pos	------------------------------------	TRUE

* reinfection compared to first sample of this patient

Negative differences to the dominant aneuploidy profile are circled in red.

Row in gray indicate strain data with aneuploidy between timeseries or aliquot sampling.
