# Supplementary material for: Diversity and Within-Host Evolution of Leishmania donovani from Visceral Leishmaniasis Patients with and without HIV Coinfection in Northern Ethiopia
Source: mBio. 2021 Jun 29;12(3):e00971-21. doi: 10.1128/mBio.00971-21 (PMC8262925; doi:10.1128/mBio.00971-21)
Supplement: FIG S7 [file mbio.00971-21-sf007.pdf]

Fig. S7 Gene copy numbers at known drug resistance loci for time series isolates.

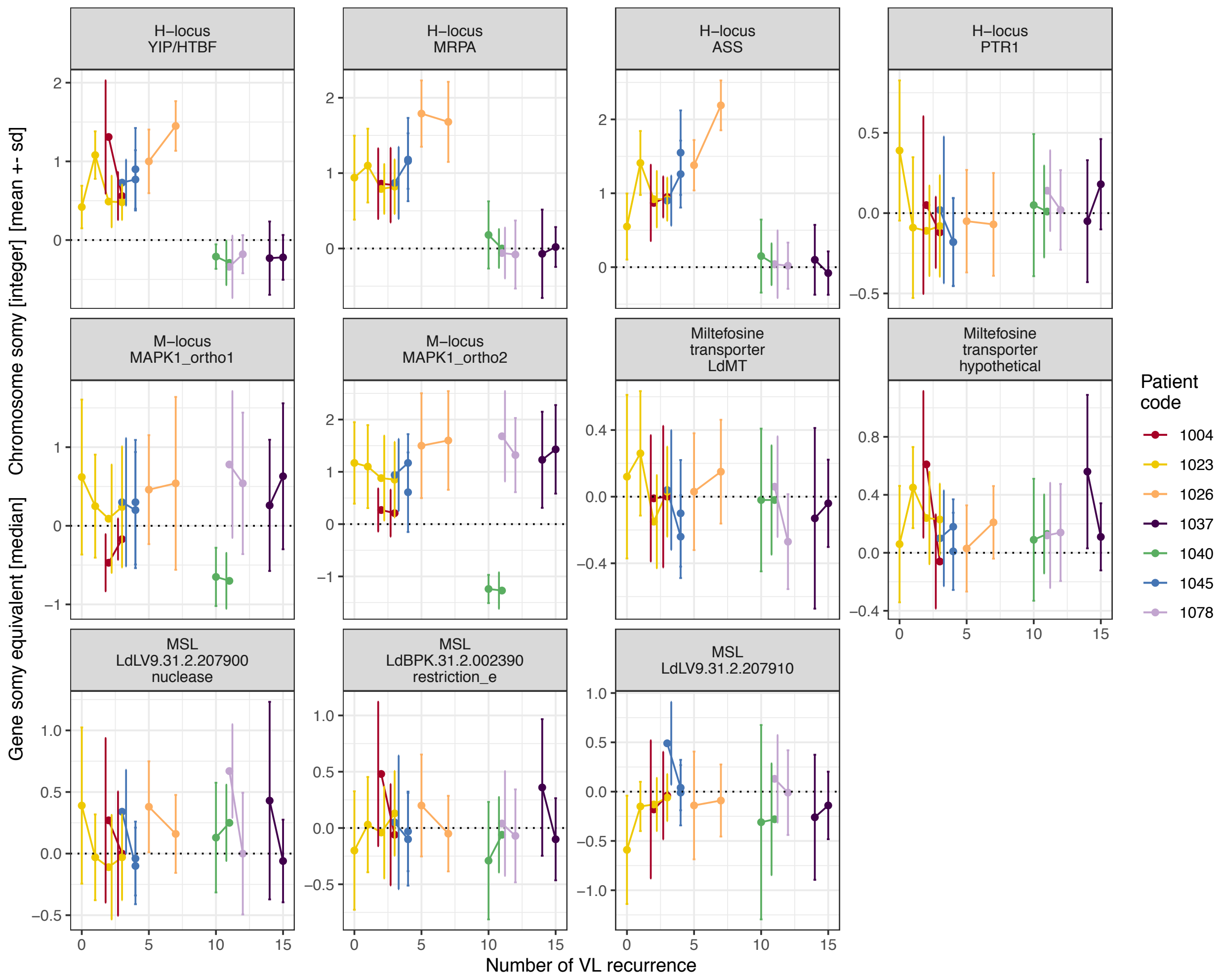

Figure S7. Gene copy numbers at known drug resistance loci for time series isolates. Gene copy numbers at drug resistance loci are shown for all the seven patients from whom we have multiple isolates sampled at different time points. Time is measured as the number of the recurrences VL episode, when the isolate was taken (0 is primary VL). Gene copy numbers are estimated as differences in somy-equivalent to the respective chromosome somy and shown are mean +/- standard deviation using individual base pair coverages within each gene.
