# Supplementary material for: Diversity and Within-Host Evolution of Leishmania donovani from Visceral Leishmaniasis Patients with and without HIV Coinfection in Northern Ethiopia
Source: mBio. 2021 Jun 29;12(3):e00971-21. doi: 10.1128/mBio.00971-21 (PMC8262925; doi:10.1128/mBio.00971-21)
Supplement: FIG S8 [file mbio.00971-21-sf008.pdf]

Fig. S8 Aneuploidy changes of parasites from patients with recurrent VL.

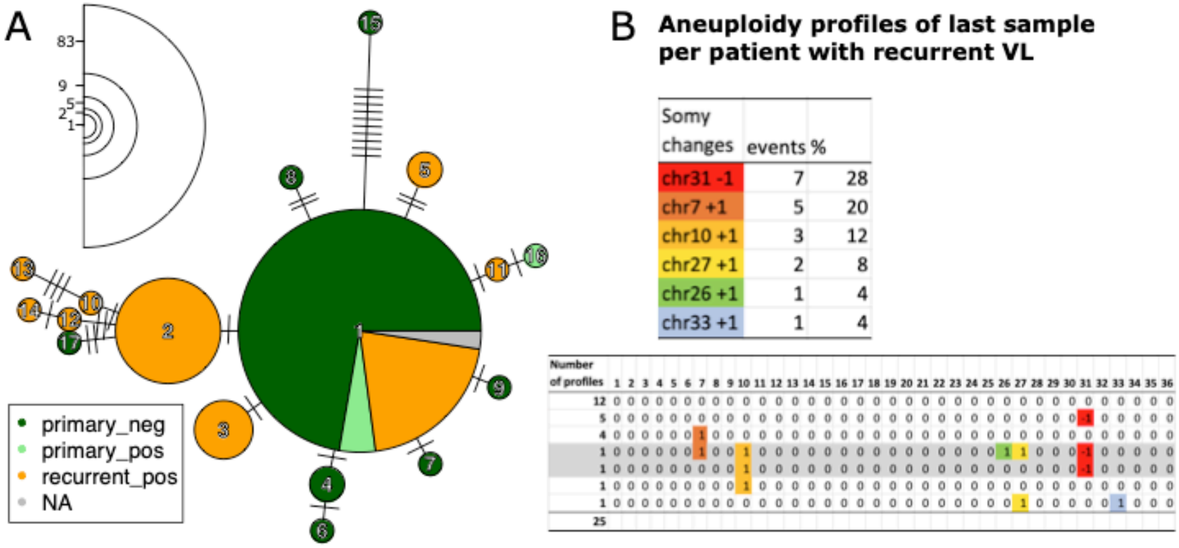

B **Aneuploidy profiles of last sample per patient with recurrent VL**

Somy changes	events	%
chr31 -1	7	28
chr7 +1	5	20
chr10 +1	3	12
chr27 +1	2	8
chr26 +1	1	4
chr33 +1	1	4

Number of profiles	1	2	3	4	5	6	7	8	9	10	11	12	13	14	15	16	17	18	19	20	21	22	23	24	25	26	27	28	29	30	31	32	33	34	35	36
12	0	0	0	0	0	0	0	0	0	0	0	0	0	0	0	0	0	0	0	0	0	0	0	0	0	0	0	0	0	0	0	0	0	0	0	0
5	0	0	0	0	0	0	0	0	0	0	0	0	0	0	0	0	0	0	0	0	0	0	0	0	0	0	0	0	0	0	-1	0	0	0	0	0
4	0	0	0	0	0	0	1	0	0	0	0	0	0	0	0	0	0	0	0	0	0	0	0	0	0	0	0	0	0	0	0	0	0	0	0	0
1	0	0	0	0	0	0	1	0	0	1	0	0	0	0	0	0	0	0	0	0	0	0	0	0	0	1	1	0	0	0	-1	0	0	0	0	0
1	0	0	0	0	0	0	0	0	0	1	0	0	0	0	0	0	0	0	0	0	0	0	0	0	0	0	0	0	0	0	-1	0	0	0	0	0
1	0	0	0	0	0	0	0	0	0	1	0	0	0	0	0	0	0	0	0	0	0	0	0	0	0	0	0	0	0	0	0	0	0	0	0	0
1	0	0	0	0	0	0	0	0	0	0	0	0	0	0	0	0	0	0	0	0	0	0	0	0	0	0	1	0	0	0	0	0	1	0	0	0
25																																				

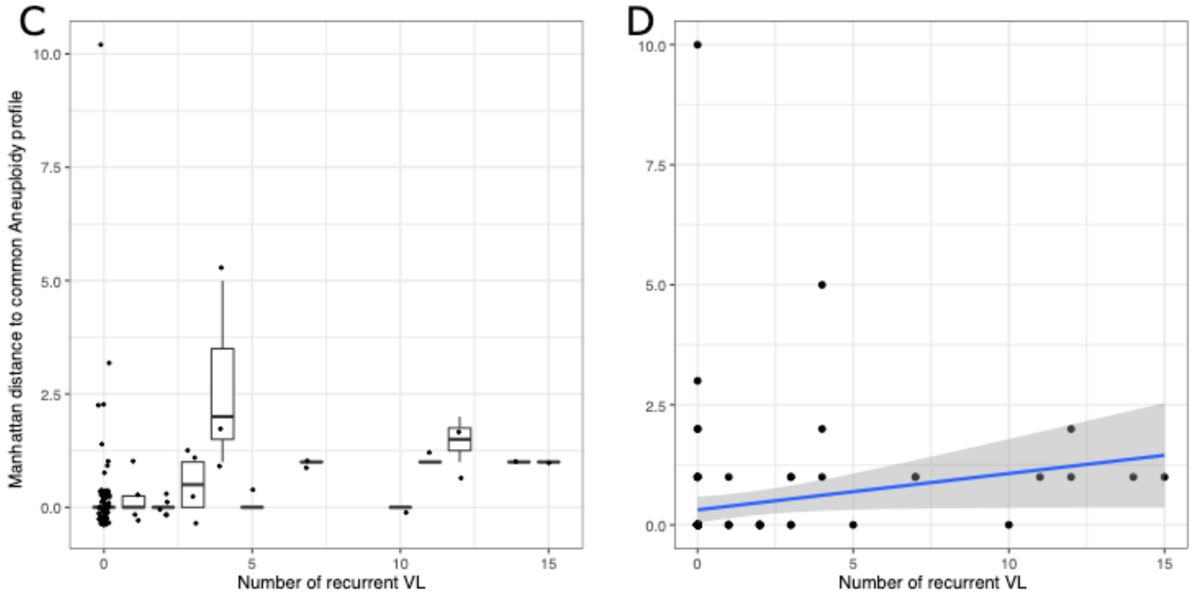

Figure S8. Aneuploidy changes of parasites from patients with recurrent VL. A) Aneuploidy profiles of all 113 parasite isolates are shown in a minimum spanning network as in figure 4 but labeled by VL and HIV coinfection status. Connections indicate profile similarity with the number of ticks indicating the sum of somy differences between connected profiles. Circle sizes represent the numbers of isolates showing a particular profile and numbers for each circle size are indicated in the left upper corner. Circles are numbered to identify each aneuploidy profile, and details of the somy patterns and abundance of each profile are listed in table 2. The largest circle (profile 1) represents the diploid condition, but with a tetrasomic chromosome 31. B) Aneuploidy profiles of the last isolate taken from each patient with recurrent VL are summarised. The first table summarises the chromosomes changed with the type of change and the frequency of occurrence out of all 25 last samples per patient with recurrent VL. In the second table the respective profiles

and number of occurrences are listed. C) & D) For aneuploidy profiles of all 96 last isolates from each patient with known relapse status Manhattan distances to the most common aneuploidy profile were calculated and shown with respect to the number of VL relapse isolated from. C) Boxplots with individual data points as jitter. D) Scatter plot with linear regression line and 95% confidence intervals.
